# Natural cell based biomimetic cellular transformers for targeted therapy of digestive system cancer

**DOI:** 10.7150/thno.75937

**Published:** 2022-10-09

**Authors:** Xiaomeng Tang, Dan Li, Yongwei Gu, Yunan Zhao, Aixue Li, Fu Qi, Jiyong Liu

**Affiliations:** 1 Department of Pharmacy, Fudan University Shanghai Cancer Center; Department of Oncology, Shanghai Medical College, Fudan University, Shanghai 200032, China.; 2 College of Pharmacy, Shandong University of Traditional Chinese Medicine, Jinan, Shandong 250355, China.; 3 Department of Pharmacy, Shanghai Proton and Heavy Ion Center, Shanghai 201315, China.

**Keywords:** Natural cell, Biomimetic cellular transformers, Digestive system cancer, Cancer targeting, Transformation

## Abstract

Digestive system cancer is the most common cause of cancer death in the world. Although cancer treatment options are increasingly diversified, the mortality rate of malignant cancer of the digestive system remains high. Therefore, it is necessary to explore effective cancer treatment methods. Recently, biomimetic nanoparticle delivery systems based on natural cells that organically integrate the low immunogenicity, high biocompatibility, cancer targeting, and controllable, versatile functionality of smart nanocarrier design with natural cells have been expected to break through the bottleneck of tumor targeted therapy. In this review, we focus on the dynamic changes and complex cellular communications that occur *in vivo* in natural cells based vehicles. Recent studies on the development of advanced targeted drug delivery systems using the dynamic behaviors such as specific surface protein affinity, morphological changes, and phenotypic polarization of natural cells are summarized. In addition to drug delivery mediated by dynamic behavior, functional “delivery” based on the natural cell themselves is also involved. Aiming to make the best use of the functions of cells, providing clues for the development of advanced drug delivery platforms.

## Introduction

Digestive system tumors mainly include esophageal cancer, gastric cancer, colorectal cancer (CRC), pancreatic cancer, hepatocellular carcinoma (HCC) and biliary tract cancer, and are the most common cause of cancer death worldwide. Its morbidity and mortality are in the forefront of malignant tumors 1. There were nearly 6 million new cases of digestive system malignancies worldwide in 2020 2. At present, the main treatment methods for early and mid-stage digestive system cancer are still surgery and postoperative adjuvant chemoradiotherapy. The most common cancer treatment strategy is chemotherapy. However, chemotherapeutic drugs often have disadvantages such as poor water solubility, low tumor targeting ability, and severe adverse reactions, which greatly limit their clinical applications 3. At the same time, because most cases are found in the late stage of the disease, the effective treatment methods are very limited, resulting in a high fatality rate 4-7. Therefore, exploring new therapeutic methods for digestive system cancer has become a hotspot of current research.

Through decades of research, nanotechnology has shown great potential in diagnosing and treating various diseases 8,9. However, the immune system distinguishes most synthetic nanocarriers as foreign substances and eliminates them 10,11. Surface modification of nanoparticles can prolong their circulation time in blood and achieve specific targeting, but this complicates their preparation 12. As a result of their dynamic interactions or information exchanges with body systems, natural cells are emerging as the optimal drug delivery platform 13.

Additionally, the cell surface has specific signaling molecules, which can drive to the disease site, and can also dynamically alter its own morphology, secretion capacity or polarization state according to endogenous or exogenous factors 14. The process of dynamic transformation can occur naturally, such as self-extrusion of red blood cells when passing through narrow capillaries, morphological changes of neutrophils passing through intercellular spaces and the release of extracellular traps, extracellular membrane vesicles with parent cell genetic material and function secreted by cells of different origins, as well as the interconversion and homeostasis of various phenotypes of macrophages *in vivo* 15. On the other hand, from the perspective of “cargo”, in addition to specific substances, various functional components of the cell itself can likewise be delivered as “cargo”, and the resulting functional transmission is also particularly important in intercellular information communication. The transmission of these functions is usually carried out by the transport of extracellular vesicles, such as information transmission driven by molecular messengers such as eicosanoids, cytokines and various chemokines. In lipid bilayers, fluidity and cytoskeletal dynamics dictate the dynamics of processes and their role in the vast majority of physiopathological processes in the cell is essential 16-19. Furthermore, the unique dynamic transformation properties of natural cells combined with advanced bioconjugation chemistry, nanotechnology and biotechnology can provide clues for the development of efficient drug delivery platforms 20,21.

**Figure 1:**
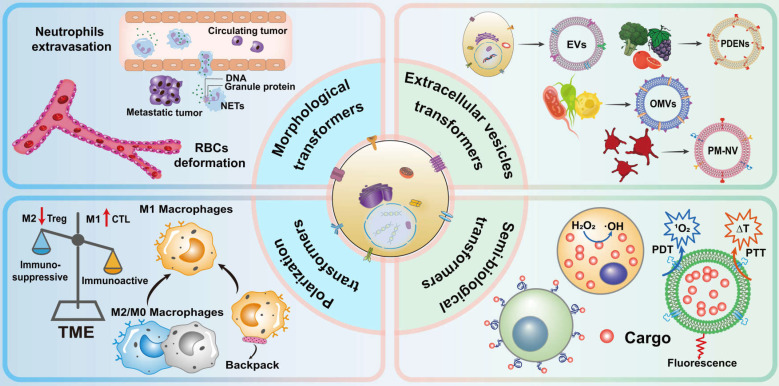
Schematic representation of biomimetic cellular transformers.

Here, we review dynamic drug delivery systems which use cells with transformable behavior instead of conventional nano- or cellular carriers. In this review, four categories of dynamic carriers of cells are covered according to their transformation behavior (**Figure 1**): morphological deformation of erythrocytes and neutrophils, vesicle secretion, macrophage polarization, and semi-biological hybrid cells, to promote further progress in this field.

## 1. Morphology deformation-based cellular carriers

Physiological and pathological changes of the body often cause changes in the number or quality (function) of blood cells, and then lead to anemia, fever, infection, bleeding and vascular embolism and other clinical symptoms, and seriously affect the communication of information and material transfer between cells 22,23. In the microvascular system, when red blood cells flow through narrow capillaries, due to shear stress, red blood cells will undergo compression and deformation in capillaries, thus promoting material exchange between red blood cells and surrounding tissues 24,25. In tumor micro-environment (TME), neutrophils release cytokines and other special messengers to enhance the ability of tumor cells to metastasize 26. Subsequently, neutrophils are activated by inflammation and TME to metastasize and accumulate in tumor tissue 27. In addition, neutrophils have multiple receptors on their surfaces, thus facilitating drug enrichment at the tumor site when neutrophils' membranes interact with chemokines and adhesion factors in the tumor microenvironment 28.

### 1.1. Red blood cell membrane

The red blood cells (RBCs) in our bodies circulate for a long time, lasting about 100 to 120 days 29. The long-term circulation effect of erythrocytes is mediated by a series of membrane proteins on the surface of the cell membrane, among which integrin-related protein CD47 plays a key role 30. CD47 is expressed on erythrocyte membrane and, as a self-protective protein, can recognize signal regulatory protein-α (SIRP-α) on macrophage membrane and send inhibitory signals to prevent phagocytosis of macrophages 31,32. In addition, the double concave disc-shaped structure of mature red blood cells is anuclear and less rigid structure, so it is easier to deform than other cells 33. When the osmotic pressure of the surrounding medium decreases, RBCs can become cup-shaped and finally form a sphere. This swelling characteristic is the key to combine RBCs with drugs or other chemicals 34. The modification of traditional nanoparticles (NPs) on the surface of red blood cell carrier is a new bionic strategy to improve the half-life of drugs *in vivo*.

The surface area of a single RBC membrane reaches 160 μm^2^, which can be used as a good drug loading platform, and the conjugation of drugs onto the RBCs membrane can reduce the damage to the structure and function of RBCs to some extent 35. It can not only load various small molecular compounds, but also macromolecular compounds such as proteins and nucleic acids, and its membrane fluidity provides better flexibility when passing through narrow capillaries 36,37. However, the high specificity of conjugating RBCs to drugs by means of membrane surface attachment leads to a lower range of potential indications. At present, the major forms of conjugation currently include the erythropoietin receptor (EPOR) mediated linkage 38, the RBCs complement receptor type I (E-CR1) mediated l linkage, the avidin-biotin mediated linkage 39, and linkage via chemical bonds. Magnani *et al*. found that the cell activity of biotin acylation by biotin *N*-hydro⁃succinimide ester (NHS biotin) is the best, the recovery rate of RBCs can reach more than 90%, about 1000 biotins are connected to each RBCs membrane, and the activity is not affected for 24 hours *in vivo* 40. Glutaraldehyde, as a commonly used cell cross-linking and fixative agent, can cross- link with proteins on the cell membrane and increase its stability, thereby avoiding RBCs hemolysis, and also slow down the drug loss from the cell, while having some targeting properties 41. Animal experiments confirmed that RBCs carrier treated with high concentrations of glutaraldehyde could target to the liver, whereas those treated with low concentrations of glutaraldehyde target to the spleen. However, the osmotic fragility of the RBCs carrier treated by this method is increased, the cell deformability is reduced, and it is difficult to pass through the narrow capillaries. Band 3 protein (Band^3^) cross-links into clusters on the membrane of senescent or degenerated RBCs, promotes the connection of IgG, and is more easily phagocytosed by macrophages. Therefore, RBCs carriers treated with Band^3^ cross-linking reagent have macrophage targeting properties. Lotero *et al*. treated RBCs carriers carrying etoposide with bis(sulfosuccinimidyl)suberate (BS^3^) and *in vitro* results showed that macrophage phagocytosis increased from 3% to 11%. The *in vivo* results showed that the treated RBCs carriers were liver-targeted due to the strong attachment of the Band^3^ to macrophages 42.

**Figure 2:**
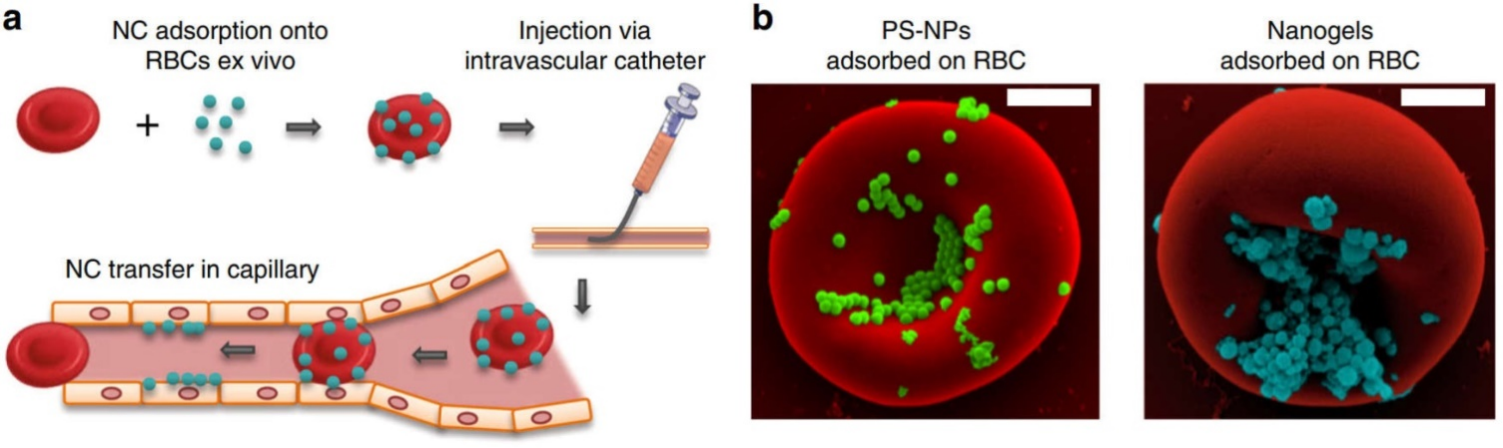
Clinically translatable nanocarriers adsorb onto red blood cells. (**a**) Procedural steps of RBC hitchhiking. NCs are first adsorbed onto the RBCs *ex vivo*. (**b**) Scanning electron micrographs of PS-NPs and nanogels attached to the surface of murine RBCs. Adapted with permission from 43. Copyright Year 2018, Springer Nature.

In addition to using cross-linking agent to couple drugs to RBCs membrane, adsorbing drugs to RBCs membrane is also an effective drug loading strategy. Brenner *et al*. developed a “red blood cell hitchhiking (RH)” technology, which adsorbs drug loaded NPs onto RBCs and then infuses them to patients through intravenous injection or arterial intubation (**Figure 2a**). After entering the blood circulation, the NPs are desorbed from the RBCs membrane under the influence of blood flow shear stress or in direct contact with endothelial cells (**Figure 2b**). The results show that RH can deliver NPs to various types of cells on the surface of capillary lumen, and the type of cells to which NPs are delivered depends on the pathophysiological environment. This new drug delivery technology can accurately deliver nano drug carriers to selected organs by placing intravascular catheters in animal or human lungs, while avoiding RBCs hemolysis or toxicity to target organs, and has good biocompatibility 43.

In addition to oxygen transport, RBCs have a variety of additional immunological related functions. Researchers have found that RBCs can bring immune complexes and bacteria from the circulatory system to liver macrophages and professional antigen presenting cells (APCs) in the spleen by capturing them on their surface. Through exploiting RBC's unique ability to present antigens in the spleen, Anvay Ukidve *et al*. developed strategies to generate cellular and humoral immune responses against antigens. Researchers designed a RBCs hitchhiking system to mainly deliver the attached NPs to the spleen but not the lungs, thereby activating cellular immunity and humoral immunity, and they termed this process as erythrocyte-driven immune targeting (EDIT). Specifically, the researchers coat surfaces of polystyrene carboxylate (PS-COOH) with ovalbumin (OVA) as a model antigen using 1-ethyl-3-(3-dimethylaminopropyl) carbodiimide (EDC) chemistry to generate protein-capped NPs attached to RBCs. Dendritic cells found all particles to be monodisperse and easily internalized and activated by them. As mentioned earlier, particulate matter can be removed from the lungs with RH technology because shear stress is experienced by RBCs undergoing deformation in the lung capillaries. Increasing the NPs to RBCs dosing ratio helps to improve the shear resistance in the lungs, thus allowing more NPs to be targeted to the spleen or other sites 44.

Despite improving the targeting and safety of drug delivery, RH strategy still has some problems: 1) NPs are not firmly connected to RBCs and easy to undergo desorption. 2) At present, the use of RH strategy to achieve the targeting of organs such as heart and brain requires the aid of arterial vascular cannulation, which is not suitable for chronic, outpatient treatment of diseases 45. 3) Construction of hitchhiking RBCs drug loading systems requires multiple steps such as RBCs isolation, *ex vivo* loading and reinfusion of RBCs, or infusion of donor RBCs, each of which can have an impact on the final yield. The adhesion of NPs to RBCs is the basis of the RH strategy, but due to the small surface area to volume ratio of RBCs, there is also the problem that the drug loading is difficult to increase 46. 4) Currently, there is a lack of studies on the distribution of NPs transferred from RBCs carrier to the vascular cells in the vasculature of addressee organs 47. 5) In the RH strategy, NPs nonspecifically attach to random components of the RBCs membrane, which may affect the normal function of RBCs. However, a spectrum of affinities to the RBCs may also arise. Understanding this aspect and designing selective coupling of NPs to carrier RBCs may enable specific transfer to intended vascular areas and cells 47,48. Although the application of RH strategy in clinic is still immature, its advantages still cannot be ignored. By adjusting the ratio of NPs to RBCs, a drug delivery system targeting different organs may be successfully constructed. Besides, using the innate immune function of RBCs to design NPs for transfer to the spleen provides an alternative format for delivering NPs to the spleen, avoiding excessive modification of NPs. The immune memory induced by EDIT can continuously drive therapeutic responses. As a general strategy, combining RH strategy with tumor immunotherapy may provide new hope for treating solid tumors such as digestive system cancers.

### 1.2 Neutrophils cell membrane

Neutrophils account for about 50% ~ 70% of the total number of leukocytes and are the most abundant leukocytes in human body 49. As the first responder of inflammatory signals, neutrophils will be “captured” by selectin (SEL) synthesized by vascular endothelial cells when flowing rapidly in the blood, making it roll slowly along the inner surface of blood vessels, detecting inflammatory signals such as complement fragment C5a and lipopolysaccharide (LPS), and then rapidly expressing integrin (INT). INT interacts with intercellular cell adhesion molecule-1 (ICAM-1) expressed on the surface of endothelial cells. Then a chemokine signal causes neutrophils to stop rolling and migrate toward the site of inflammation by passing through the vascular endothelium 50. Although the spacing between endothelial cells is not enough for a cell to pass through, neutrophils can be deformed by squeezing itself through the cell gap. This is also known as leukocyte extravasation. However, excessive neutrophils infiltration is also the main reason for the continuous deterioration of some diseases. Researchers have found neutrophils infiltration in a variety of tumor models 51-55. Chemokines and cytokines produced by tumor cells and the surrounding microenvironment will actively recruit neutrophils. Neutrophils express chemokine receptors CXCR1 and CXCR2 56, and tumor cells express the ligands of these receptors, thereby promoting the recruitment of tumor associated neutrophils. Neutrophils can also interact with circulating tumor cells (CTCs) through adhesion molecules on cells and neutrophil extracellular traps (NETs) to promote tumor metastasis 57,58. Therefore, neutrophils can be used as a good provider of tumor targeted cell membrane biomimetic materials 59.

**Figure 3:**
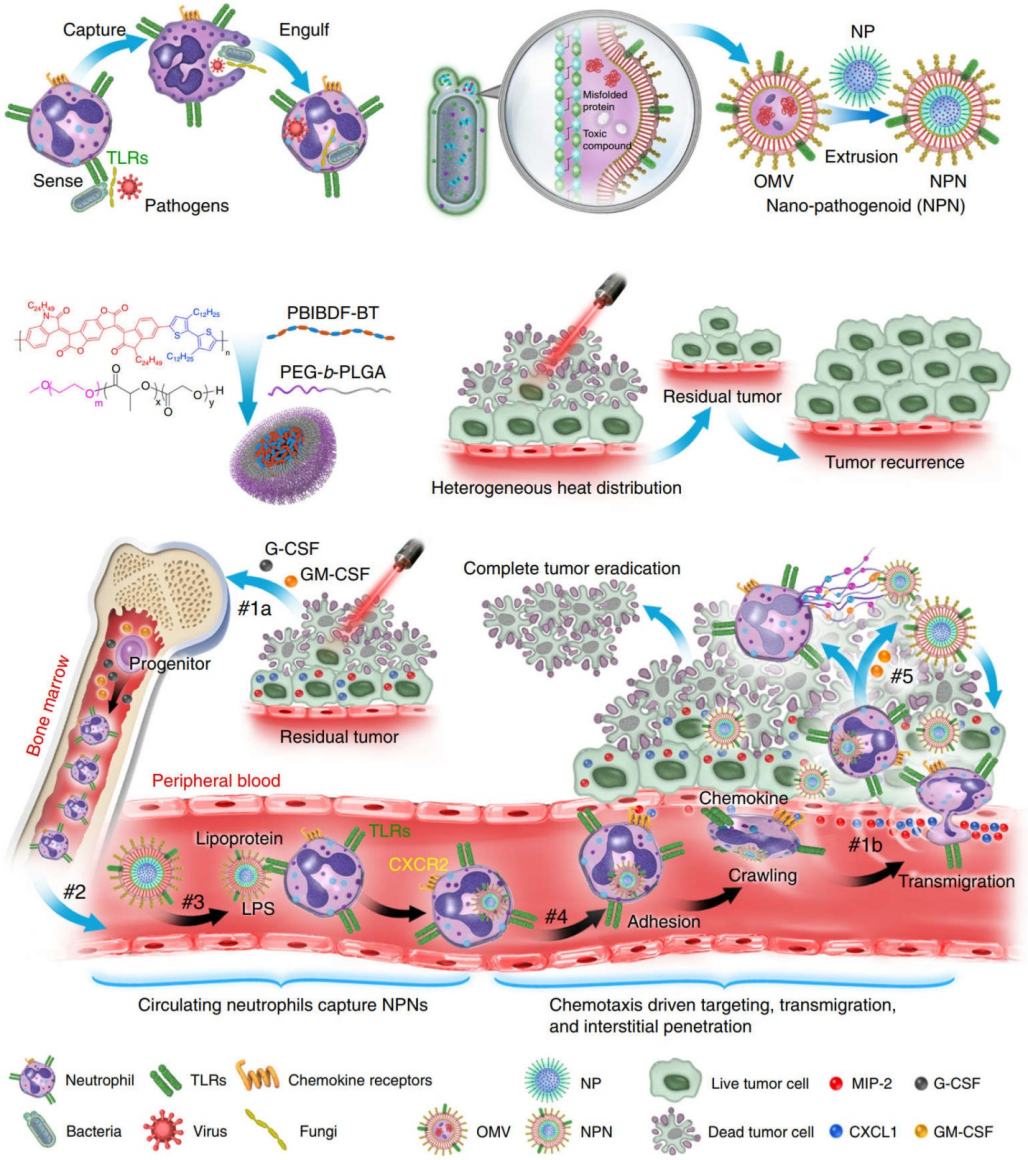
Schematic illustration showing the chemotaxis-driven delivery of NPNs for complete eradication of tumors post-phototherapy. **#1a** The released G-CSF and GM-CSF increased neutrophil production from bone marrow. **#1b** The released CXCL1 and MIP-2 broadcasted the location of the inflamed tumor. **#2** Neutrophils entered the blood circulation and encountered the injected NPNs. **#3** Neutrophils sensed NPNs with the recognition of LPS and lipoprotein by TLRs and subsequently engulfed them. **#4** Neutrophils laden with NPNs were recruited into the tumor site in response to the chemokine gradient through the following cascade: adhesion, crawling and transmigration. **#5** NPNs were released from neutrophils to kill tumor cells along with the formation of NETs in the inflamed tumor. Adapted with permission from 61. Copyright Year 2020, Springer Nature.

The use of neutrophils to deliver drugs or NPs generally takes two forms. The first is to assemble NPs into neutrophils *in vitro*. However, in practical applications, due to the short lifespan (~ 7 h) of neutrophils, intracellular degradation of drugs, insufficient number of obtained cells, and the potential for *in vitro* contamination, these methods are not effective 60. The second is to use NPs to “hitchhiking” neutrophils *in situ* (i.e., during blood circulation). Although this approach is more beneficial to clinical practice, it is crucial that NPs are designed to have high affinity for neutrophils. Li *et al*. constructed a strategy based on pathogen mimicking nano-pathogenoids (NPNs) to “hitchhiking” circulating neutrophils, and combined photothermal therapy to enhance the recruitment of neutrophils by NPNs from the blood circulation and the ability to eliminate residual tumor cells 61. The researchers first constructed NPNs by coating bacteria-secreted outer membrane vesicles (OMVs) collected from *E. coli* on NPs by extrusion method. During photothermal therapy (PTT), the chemokines produced stimulate the neutrophils and inform them of the tumor's location. Pathogen-associated molecular patterns (PAMPs) on NPNs are sensed and recognized by pattern recognition receptors (PRR) on neutrophils to “hitchhiking” circulating neutrophils, on the basis of which biological barriers are overcome and actively accumulate at the tumor site. After NPNs enter tumor tissue, in the process of forming neutrophil extracellular traps (NETs), NPNs are released from neutrophils when plasma membranes are destroyed and internalized by tumor cells to exert anti-tumor effect (**Figure 3**). Real-time intravital microscopy was used to examine the migration patterns of neutrophils in PTT-treated tumors, thus confirming that chemotaxis of neutrophils to tumors can overcome certain biological drug delivery barriers. Within 2 hours after PTT, a large number of neutrophils aggregated into tumors. In enlarged region I, the shape of neutrophils changed from oval to irregular as it moved slowly along blood vessels, sometimes adhering to vessel walls. In enlarged region II, when neutrophils are elongated, they can move more freely in small capillaries, allowing them to be evenly distributed throughout the tissue. In addition, neutrophils on blood vessels also enter the interstitium by elongating and squeezed through endothelial cells. The above results all indicated that the recruited neutrophils could cross the vascular barrier in a dynamic deformation manner and increase the distribution in the tumor tissue interstitium. The researchers further induced an inflammatory environment with PMA medium and verified that the encapsulated drugs could be released from neutrophils. The mechanism of its release lies in the dynamic deformation of activated neutrophils and eventually form NETs, which indicates the plasma membrane of neutrophils has disintegrated, thereby leading to the final release of the drug. Further results from *in vivo* experiments confirmed that PTT-induced inflammation led to the release of encapsulated NPs by NETs, followed by its eventual endocytosis by tumor cells. Usually, as a result of the limited penetration of light through tumor tissue, the uneven distribution of heat simply retards tumor growth but does not eliminate it completely, the result was a recurrence and metastasis of the tumor. By combining PTT and chemotherapeutic drugs, NPNs dislodge circulating tumor inducing neutrophils *in situ*, thereby eradicating tumors in all treated mice. This method also provides new ideas for improving nanomedicine delivery and tumor therapy 61.

### 1.3 Stem cell membrane

Stem cells are a type of pluripotent cells with self-replication ability. Studies have shown that cytokines and chemokines secreted by tumor cells can induce the enrichment of stem cells into tumor tissue 62. Therefore, many types of stem cells, such as bone marrow-derived mesenchymal stem cells (BMSCs), can be used to construct tumor targeted nanomedicines. MSCs have a strong ability to orientate malignant lesions, that is, the homing characteristics of stem cells. When ischemic, hypoxic, or injured, body endogenous or exogenous stem cells have the trait of directional migration toward the site of injury and distribution 63. The homing mechanism of stem cells is a multi-step and coordinated process, which mainly involves chemokine receptor interactions, adhesion of endothelial cells, and so on 64. Chemokines released by tumor and tumor associated stromal cells attract BMSCs with corresponding receptors against a concentration gradient to reach the tumor site 65. After stem cells are mobilized and migrated, their vascular cell adhesion molecule (VCAM) and intercellular adhesion molecule (ICAM-1) interact with endothelial cells to allow stem cells to adhere to the capillary wall for targeting across the endothelial cell layer to the tumor site by extrusion deformation 66. MSCs are easy to isolate and culture *in vitro*, and encapsulating nanoparticles with stem cell membrane can endow the nanocarrier with active targeting property, which can specifically accumulate in the tumor site, reduce the damage to the body's normal tissues and improve the therapeutic efficacy.

To reduce the systemic toxicity of doxorubicin (DOX), Liu *et al*. constructed a MSCs membrane encapsulated DOX superparamagnetic iron oxide (SPIO) NPs for colorectal cancer treatment. DOX-SPIO@MSCs with the biological function of MSCs membrane, thus achieving highly efficient delivery of intracellular drugs, prolonging serum half-life, and actively targeting to the tumor site. Compared with free DOX and DOX-SPIO, DOX-SPIO@MSCs can not only avoid the premature clearance of drugs by the mononuclear phagocyte system, but also have stronger targeting to the MC38 xenograft tumor. The anti-tumor treatment efficiency was significantly improved without obvious systemic toxicity 67. Compared with BMSCs, human umbilical cord derived MSCs are popular sources because of their stemness, ease of acquisition, high rate of proliferation, and provision of abundant plasma membranes. Yang *et al*. noted that PLGA NPs encapsulated by umbilical cord derived MSCs could enhance the uptake of DOX, and the results showed that the uptake efficiency of membrane coated NPs by tumor cells was 3 times higher than that of membrane uncoated NPs. Moreover, encapsulation of umbilical cord derived MSCs improved the release efficiency of DOX at acidic microenvironment and inhibited MHCC97H subcutaneously tumor growth in nude mice with liver cancer 68. Notably, stem cells exhibit different growth impacts for different tumor models, suggesting that the targeting ability of stem cell membrane biomimetic NPs may be tumor-specific and not applicable to all tumor types 69. At the same time, it is still necessary to further confirm whether the stem cell carrier has the risk of promoting tumor growth or even metastasis, so its clinical application still needs further exploration.

## 2 Cellular dynamic release-based delivery

Under physiological and pathological conditions, almost all kinds of microorganisms or mammalian cells will dynamically release extracellular vesicles (EVs). EVs are diverse membranous vesicles derived from endosomes and cell membranes released by cells into the extracellular environment 70. As early as 1976, Allan *et al*. found that RBCs release EVs from their surface 71. EVs can not only participate in the transport of biomolecules such as lipids, proteins and RNA between cells, but also mediate the transmission of information between cells in biological system 72-74. EVs can be divided into exosomes, microvesicles (MVs), apoptotic bodies, synaptoneurosomes and other types 75,76. The diversity of EVs stems from its different production mechanisms 77. Because EVs mediate the communication between cells, have the ability of material loading and certain targeted homing ability, and have high biocompatibility and can exist stably *in vivo*, research on EVs and drug delivery has increased gradually in the recent years 78. Therapeutic drugs can be loaded into EVs in various ways. A comparison of the advantages and disadvantages of each drug loading method is shown in **Table 1**. In addition, EVs have lipid bilayers, so lipid-soluble molecules can be anchored and inserted into the membrane. Combined with cell membrane surface modification technology, functionalized EVs can be obtained, which further expands the application of EVs as drug carrier 79. This part will focus on the bionic drug delivery system based on the dynamic release of these cells.

### 2.1 Extracellular vesicles of animal origin as dynamic carriers

EVs is a highly heterogeneous vesicle 91. The release of EVs is considered to be an important regulator of cell-cell interaction and has a wide range of physiological functions 92. It can be used as antigen to bind to receptor cells, and can also be used as carrier to deliver functional substances to receptor cells, such as protein, nucleic acid, DNA, mRNA and non-coding RNA. Receptor cells can interact with EVs through several active uptake modes, including membrane fusion, receptor-mediated endocytosis, endocytosis and phagocytosis 93. The characteristics of EVs are different depending on the source cells 94. The EVs derived from mesenchymal stem cells (MSCs) can transfer many components such as genetic material and drugs. Their homing ability to inflammatory sites can also have a variety of effects on tumor growth 95. Embryonic stem cells (ESCs) can remain undifferentiated for a long time in the process of *in vitro* culture, so as to generate EVs with stable characteristics, which can be used as drug carriers for the development of new cancer therapeutic drugs. Macrophage derived EVs can penetrate the blood-brain barrier, interact with and accumulate in cancer cells 96. Tumor derived EVs have also attracted the attention of many researchers. Its tumor targeting homing property enables drugs to reach cancer cells effectively, which not only protects therapeutic drugs from degradation, but also has low immunogenicity 97. Dendritic cell-derived EVs contain a variety of antigen-presenting molecules and costimulatory molecules, which can activate T cells, enhance the function of natural killer cells, and promote the eradication of tumors 98. In addition to the above characteristics, the main advantage of EVs as drug carriers is that they can effectively load therapeutic drugs without significantly changing membrane structure and surface proteins, and have stability, biocompatibility, targeting and the ability to overcome natural barriers in blood circulation. They also have immune tolerance and can overcome the limitations of synthesizing NPs. Based on the above advantages, extracellular vesicles have become a promising tool for drug delivery 92,99-101.

Inspired by EVs mediated intercellular communication, Silva *et al*. constructed a hybrid carrier consisting of macrophage derived EVs encapsulating different therapeutic agents and iron oxide NPs to achieve spatial control of drug delivery. Studies have found that under a magnetic field, the uptake of EVs by tumor cells can be dynamically regulated and spatially controlled, and that magnetic targeting enhances tumor cell death with attenuated toxic side effects 102.

**Figure 4:**
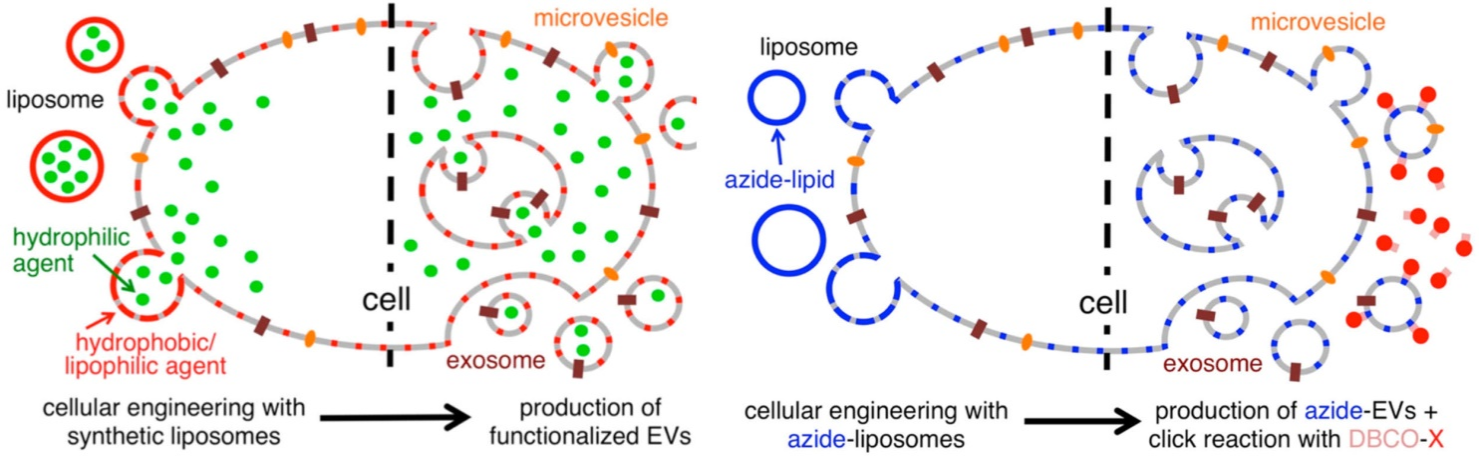
Liposome-based cellular engineering for efficient intracellular packaging of cargo in extracellular vesicles. Adapted with permission from 108. Copyright Year 2016, American Chemical Society.

With endogenous exosomes, immune reactions can be avoided. Nevertheless, the efficiency of exosome delivery depends on both the parent and the recipient cells, and native exosomes have difficulties locating their target cells 103. To meet the experimental needs, the technology of constructing engineered exosomes by surface modification has emerged 104. To precisely target therapeutic drugs to lesions, chemical attachment of targeting peptides, genetic engineering of exosome membranes, magnetic nanoparticle technology, electrostatic interactions and post-insertion have become a common method in recent years 105,106. In order to produce the precise and improved therapeutic effect, Tamura R *et al*. modified exosomes with cationic amylopectin to target asialoglycoprotein receptors on hepatocytes 107. As an alternative method, Lee J *et al*. treated parental cells with azide membrane-fused liposomes (MFL) to create azide exosomes that were then conjugated with a tumor targeting peptide conjugate DBCO-CGKPK via bioorthogonal reaction 108. MFL exogenously incorporated into exosomes for surface modification does not damage membrane proteins critical to the exosomes' biological function (**Figure 4**).

### 2.2 Extracellular vesicles of plant or microbial origin as dynamic carriers

The majority of studies at present focus on EVs derived from animal cells. However, the production efficiency of EVs derived from animal cells is low, and only a few micrograms can be obtained in billions of cells 109. Therefore, it is usually necessary to culture a large number of cells to produce enough EVs for *in vitro* and *in vivo* experiments 109, resulting in expensive experiments. Compared with EVs of animal origin, a major benefit of EVs derived from plants or microorganisms is that they can be produced in larger quantities as well as having a higher economic benefit. Not only that, plant-derived exosome-like nanoparticles (PDENs), which are often enriched in various bioactive lipid, protein, RNA, and other components, are natural nano-formulations that are proven to have significant regulatory roles in cancer, immunomodulation, and antiviral versus antioxidant effects 110. For example, Zhu *et al*. found that after oral administration of ginger derived EVs in mice, they mainly accumulated in the liver and mesenteric lymph nodes, and could inhibit ROS production when acting on hepatocytes 111. Citrus derived EVs can participate in regulating the gene expression of tight junction proteins, thereby restoring intestinal barrier function 112. Grape derived EVs accelerate the repair of intestinal mucosa epithelium by regulating the expression of genes that promote the growth and proliferation of intestinal stem cells through Wnt signaling pathway 113. Broccoli derived EVs can target dendritic cells (DCs) to activate AMP-activated protein kinase (AMPK) in DCs, thereby reducing the release of IFN-γ and TNF-α. It can prevent the activation of intestinal DCs and improve the symptoms of DSS induced colitis in mice 114. Lemon derived EVs can inhibit the proliferation of cancer cells by promoting tumor necrosis factor related apoptosis inducing ligand (TRAIL) - mediated apoptosis and inhibiting the secretion of vascular endothelial growth factor A (VEGF-A), IL-6 and IL-8 115. Lentinus edodes derived EVs can alleviate D-galactosamine/lipopolysaccharide induced liver injury in mice by inhibiting the activation of NLRP3 inflammasome 116. At the same time, in addition to their inherent therapeutic potential, source plants possess low toxicity and safety characteristics, making them ideal vehicles for delivering drugs and genes, with broad application prospects 117.

**Figure 5:**
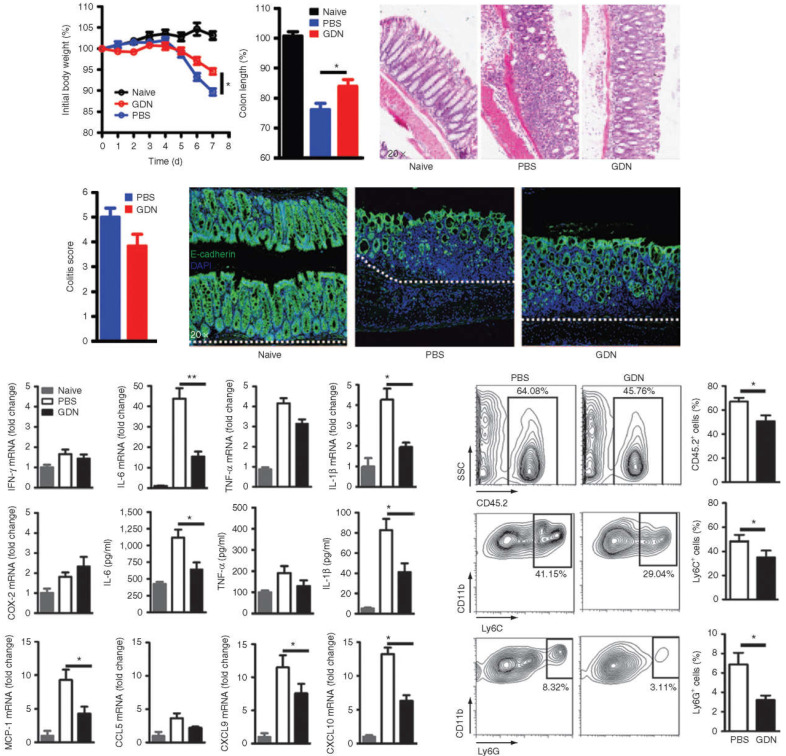
Grapefruit-derived nanoparticles (GDN) pretreatment ameliorates DSS-induced colitis in mice. C57/B6 mice were treated with either PBS/DSS or GDN/DSS. Adapted with permission from 119. Copyright Year 2014, Elsevier.

PDENs have been successfully reported for oral colon-targeted drug delivery 118. In addition, studies have also shown that PDENs have good tolerance to digestive enzymes like pepsin, intestinal pancreatin and bile. It allows the entrapped drugs to be delivered intact to the colonic site and exert their effect, thus avoiding decomposition of drugs by the gastric or intestinal environment 110. However, researchers have not yet elucidated how PDENs can still maintain drug loaded integrity while defending against the harsh gastrointestinal environment. A study constructed methotrexate loaded PDENs (GDNs) for targeting intestinal macrophages. After oral administration of the drug loaded PDENs, the results showed that in the intestinal lamina propria, these PDENs are highly efficient in targeting F4/80+ macrophages via micropinocytosis and clathrin-dependent cellular uptake pathways. After being internalized by macrophages, encapsulated methotrexate was dynamically released. The results showed that pretreatment with GDNs could effectively prevent weight loss and intestinal segment shortening caused by DSS induced colitis (**Figure 5a, b**). After treatment with GDNs, the severity and pathological score of colitis in mice decreased (**Figure 5c, d**). The expression of E-cadherin, IL-6 and IL-1β in colon epithelial cells were significantly reduced (**Figure 5e, f**). The mRNA levels of chemokines MCP-1, CXCL9 and CXCL10 were also reduced (**Figure 5g**), and the inflammatory neutrophil infiltration of CD11b^+^Ly6C^high^ was significantly reduced (**Figure 5h, i**), suggesting that GDNs could enhance the anti-inflammatory capacity of intestinal immune cells by playing the role of immune regulators 119. It has been reported that curcumin can be delivered to colon tissue using PDENs, and the PDENs enhanced both the stability and blood level of the drug, resulting in its specific drug delivery to inflammatory cells 120, offering new treatment options for colon cancer. In response to relevant studies, the James Graham Brown Cancer Center initiated two phase I clinical trials to test the therapeutic efficacy of PDENs in head and neck cancer and colon cancer (NCT01668849, NCT01294072). In addition, clinical studies on PDENs, multiple clinical trials on cell-free immunotherapy for cancer are also underway, with a view to developing effective cancer vaccines in the future 121.

Outer membrane vesicles (OMVs) are a type of membrane-shaped structure originated from the bacteria's outer membrane and mainly produced by Gram negative bacteria 122,123. OMVs contain a variety of components of bacterial origin, including enzymes, virulence factors, bacterial specific antigens, and various PAMPs, such as lipopolysaccharide (LPS), making them especially useful for the development of bacterial vaccines and adjuvants 124,125. In addition, OMVs may also have antitumor properties that have gradually gained attention 126. OMVs can not only induce powerful tumor immune effects through bacterial-derived proteins, but also encapsulate therapeutic drugs using their own structures to achieve PAMPs-mediated cell specific targeting and improve drug accumulation at target sites 127.

**Figure 6:**
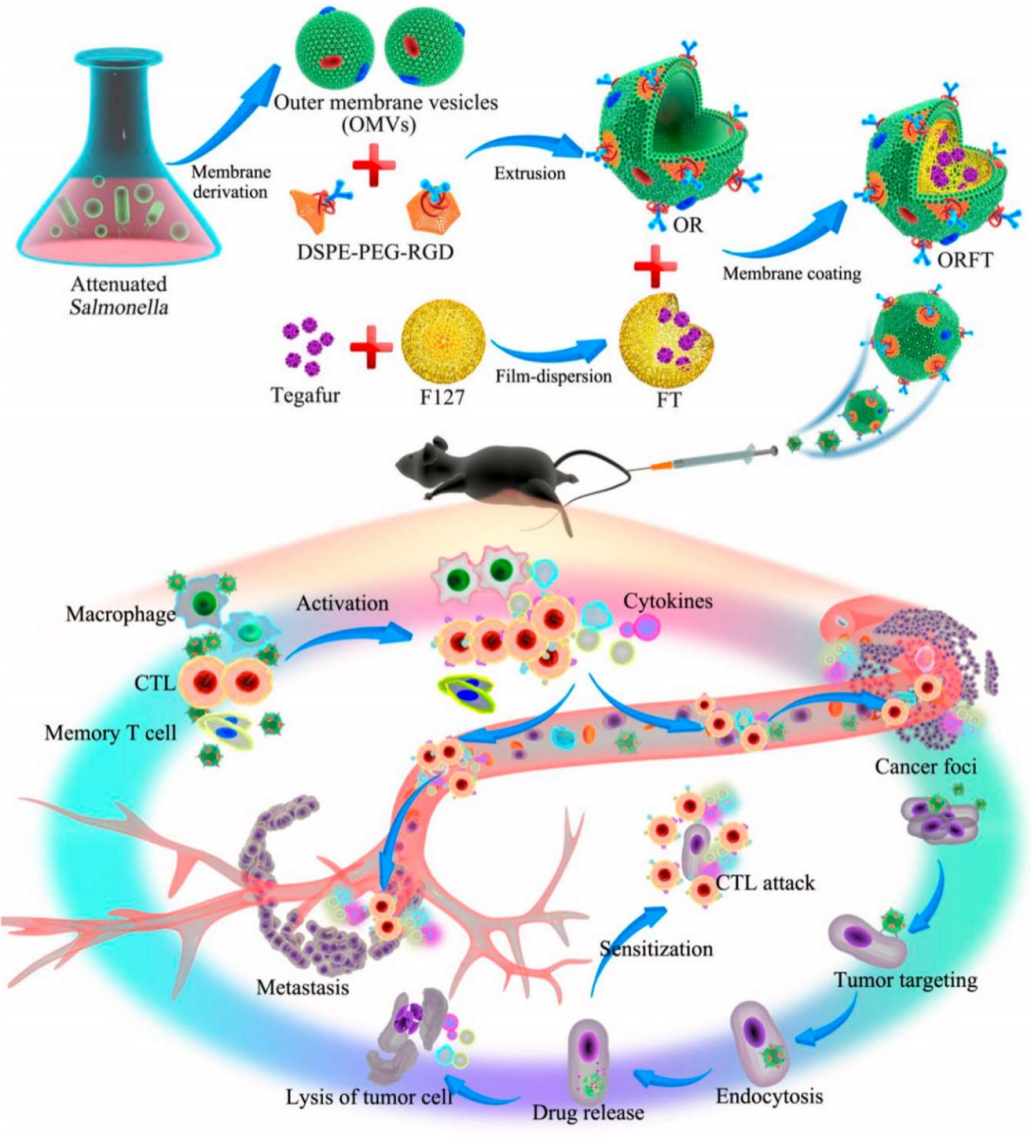
Schematic Illustration of the Bioengineering Process of Functionalized OMV-Coated Polymeric Micelles and Their Proposed Mechanism of Immunotherapy for Protective Immunity, Cancer Treatment, and Metastasis Preventiona. Adapted with permission from 128. Copyright Year 2020, American Chemical Society.

In order to relieve the state of tumor immunosuppression, combining the anti-tumor immune response mediated by OMVs with chemotherapeutic drugs is a more effective approach. By combining immunotherapy and OMVs, additional potential for tumor immunotherapy can be enhanced to prevent tumor recurrence and metastasis and complete eradication of tumors. The detoxified OMVs were modified by Ping *et al.* by adding polyethylene glycol (PEG) and the Arg-Gly-Asp (RGD) peptide to increase blood circulation and improve tumor targeting. On top of the tegafur-loaded NPs, modified OMVs were coated, and the resulting NPs had three antitumor functions (**Figure 6**). As a first step following systemic injection, innate immune cells are activated, followed by an enhanced tumor accumulation in response to the EPR effect and peptide targeting by RGD. After being taken up by tumor cells, OMVs dynamically released tegafur, which enhanced the sensitivity of cancer cells to cytotoxic CD8^+^ T cells and eliminated myeloid-derived suppressor cells (MDSCs), thereby synergistically enhancing the immunotherapy ability of OMVs 128. Notably, OMVs-coated NPs were not harmful to major organs. Serum cytokine levels increased rapidly within a few hours of injection and rapidly decreased within 24 hours, demonstrating the safety of this drug delivery vehicles.

The safety of using OMVs for cancer treatment is a major concern. Although many studies have demonstrated that the use of attenuated or detoxified OMVs as drug delivery carriers are safe in mice 129, humans are several orders of magnitude more sensitive to endotoxin than other mammals, such as mice 130. There is still no relevant report on whether the human body can tolerate OMVs. The LPS of the cell wall of gram-negative bacteria is not only highly carcinogenic but can also cause fatal septic shock 129. In order to further improve the safety of OMVs as drug carriers, current studies usually use the OMVs of attenuated or avirulent bacteria, and suppresses the expression of bacterial LPS through gene mutation or makes the cytoplasmic A in LPS insufficiently acylated, followed by genetic and surface modification to target the cells of interest. There have been studies to engineer avirulent* Staphylococcus aureus* (*S. aureus*) mutants by genetic engineering that produce avirulent cytolysins, and OMVs containing avirulent cytolysins that are immunogenic and nontoxic are able to protect mice against S. aureus 131. Gujrati *et al*. used genetic engineering to modify the parental *Escherichia coli* (*E.coli*) to reduce the cytotoxicity of OMVs, expressed the tumor targeting ligand HER2 on their surface, and loaded siRNA to inhibit the activity of overexpressed spindle kinesin within tumor cells. Consequently, cell proliferation is inhibited and apoptosis is induced. The experiments showed that Affi_HER2_OMV^siRNA^ was not significantly cytotoxic and could aggregate at the tumor site with high specificity, effectively inhibiting tumor growth 132.

### 2.3 Platelets as dynamic carriers

Megakaryocytes produce small, anucleate cells called platelets, whose lifespan is about 8 ~ 10 d, and their main function is hemostasis, regulating inflammation, and promoting thrombosis 133. Aside from their traditional functions, platelets can also play other roles such as regulating immunity and communicating with other cells and tissues in the blood vessels. Key to the role played by platelets is their activation, which upon activation enables the dynamic release of granules (platelets contain 3 types of granules: α particles, δ particles and lysosomal particles) and EVs (microparticles 100 nm ~ 1 mm and exosomes 40 ~ 100 nm in diameter). It is possible that platelet communication with other cells in the blood vessels is achieved through microparticles. Genetic material (miRNA, mRNA, and so on), enzymes, proteins, and small molecules within these microparticles can affect cells in the vasculature and alter their functions 134,135. Because platelet microparticles and exosomes differ in their source and specific protein composition, they may have different functions, with exosomes play the function of extracellular communication, platelet microparticles play the role of procoagulant activity and intracellular communication that can transfer their cargo (cytoplasm or membrane proteins, mRNA, and non-coding RNA) to target cells. This explains part of the role of platelet EVs in inflammation, thrombosis, immune regulation and biological information transmission 136,137.

Studies have shown that platelets are involved in tumor development, including tumor growth, tumor cell extravasation, and metastasis, by protecting tumor cells from the host immune system, whereas platelet effects on local and distant tumor host interactions may be mediated through the secretion of large amounts of platelet microparticles and exosomes 138,139. *In vivo* studies have shown that platelet microparticles can promote the growth of primary tumors, stimulate vascularization, and promote the formation of distant metastases 140. Levels of platelet microparticles are markedly elevated in a large subset of malignancies. Colon cancer patients are often accompanied by altered coagulation activity, and stage III/IV colon cancer patients have significantly increased levels of platelet microparticles in the systemic circulation, greatly shortened clotting time, and increased fibrin production, resulting in a hypercoagulable state in the blood of colon cancer patients, so platelet microparticles are potential targets for preventing coagulation in colon cancer 141.

**Figure 7:**
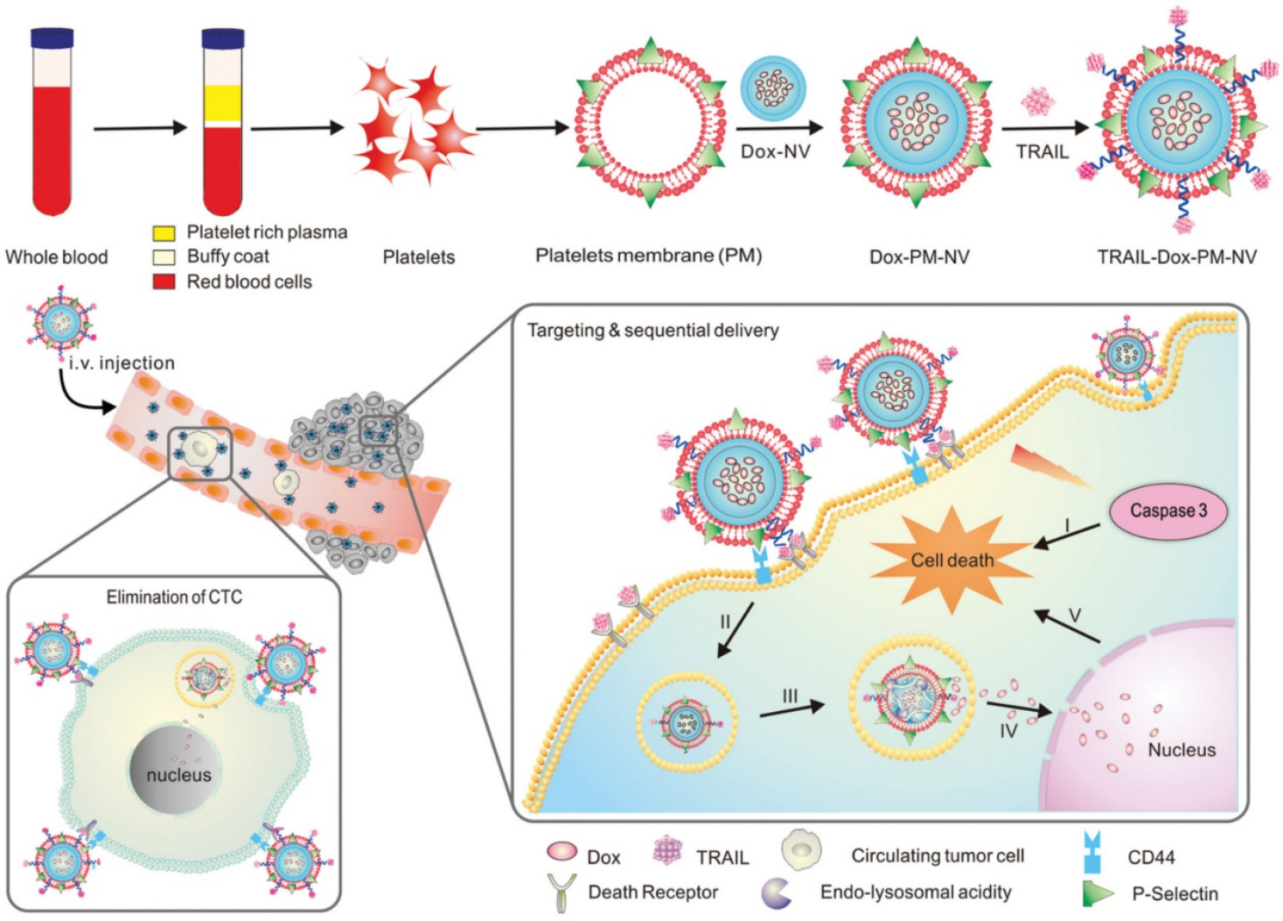
Schematic design of drug-loaded PM-NV for targeting and sequential drug delivery. Adapted with permission from 146. Copyright Year 2015, Wiley-VCH GmbH.

A great deal of attention has been paid in recent years to the interactions between platelets and CTCs in the blood 142, and the aggregation of platelets surrounding CTCs helps them survive in the bloodstream and invade new tissues 143. The high affinity between P-selectin on platelets and CD44 receptors on tumor cells explains this specific aggregation. Therefore, platelets are more likely to target CTCs when P-selectin binds specifically to CD44 144,145. To deliver functional small molecules with precise targeting, Hu *et al*. created platelet-mimicking nanovehicles (PM-NV) 146. PM-NV consists of a small molecule drug loaded nanogel inner core and a platelet membrane based outer shell. Meanwhile, TRAIL was modified on the surface of platelet membrane, and DOX was loaded into PM-NV to prepare TRAIL-DOX-PM-NV. During *in vivo* experiments, PM-NV was found to contain a large amount of platelet-derived “self-recognition” proteins, which minimizes *in vivo* immunogenicity and prolongs circulation time. CD44 molecules are present on tumor cell membranes and can be bound by P-selectin carried on platelet membranes. This allows TRAIL-DOX-PM-NV to target tumor cells and accumulate at the tumor site. Meanwhile, TRAIL-DOX-PM-NV aggregation on tumor cell surfaces encourages TRAIL interaction with the membrane and signals exogenous apoptosis. More importantly, when TRAIL-DOX-PM-NV was internalized by cells, the acidic environment of lysosomes would mediate TRAIL-DOX-PM-NV to dynamically release DOX, allowing DOX to accumulate in tumor cell nuclei and synergistically induce tumor cell apoptosis (**Figure 7**). In addition, the binding of P-selectin to CD44 can eliminate CTCs, preventing tumor metastasis. Based on platelet and tumor cell specific affinity, this study successfully developed platelet membrane modified nanomedicines that delivered TRAIL and DOX to tumor cells.

Primary amine or thiol residues on the surface of platelets enable chemical attachment to NPs or biomolecules, followed by dynamic release of drug loaded microparticles via activation 147,148. Kailashiya *et al*. 12 constructed platelet-transformed drug loaded platelet-derived microparticles (PMPs). This biomimetic particle has good biocompatibility and natural targeting, can carry a variety of drugs, has good stability, and can be obtained in large quantities in a short period of time. Compared with free drugs, PMPs are more toxic to cancer cells and less prone to extravascular escape 149.

## 3 Macrophages transformation

The body's immune system functions as an immune surveillance that can recognize and clear mutant cells from the body 150. However, tumor cells evade immune surveillance and immune killing by reducing immunogenicity and enhancing immune tolerance, leading to a loss of balance in the interaction of the tumor with the immune system 151. Tumor cells establish an immunosuppressive TME by secreting immunosuppressive factors and recruiting immunosuppressive cells, which further suppress antitumor immune responses, thus leading to uncontrolled tumor growth and ultimately life-threatening health. Tumor associated macrophages (TAMs) are important components of the immunosuppressive TME 152. The classical phenotype of macrophages is divided into M1, which is pro-inflammatory and anti-cancer, and M2, which is pro-cancer. TAMs are generally M2 type, therefore, polarizing M2 type TAMs to M1 type is a feasible scheme to restore antitumor immune responses 153.

### 3.1 Macrophage polarization

Peptides targeting M2 macrophages can be used to modify carriers capable of delivering drugs that promote macrophage transformation to achieve TAMs-specific regulation. Signal transducer and activator of transcription 6 (STAT6) and nuclear factor kappa-B (NF-κB) promote M2 polarization of macrophages through different mechanisms. Therefore, inhibition of these two signaling pathways may lead to a phenotypic transformation in macrophages. Xiao *et al*. 154 constructed a nanomedicine with dual characteristics of TME response and active targeting. The M2 targeting peptide was first masked with pH-sensitive PEG, and then the targeting peptide was used to modify the STAT6 inhibitor AS1517499 (AS) and the inhibitor of nuclear factor kappa-B kinase β (IKKβ) siRNA nanomedicines to obtain nanocomplexes. After the nanocomplex reaches the tumor, the PEG protective shell falls off under the weak acid condition of TME, exposing the M2 targeting peptide, and the loaded drug is specifically delivered to M2 macrophages, which induces the phenotypic polarization of M2 macrophages at the same time, reduce the occurrence of adverse reactions, and provide safe and effective immunotherapy. In addition, based on specific proteins on the surface of macrophages, Kulkarni *et al*. designed a protein that can specifically bind to SIRP-α and inhibit the colony stimulating factor-1 receptor (CSF-1R) through computational biology. The supramolecules can self-assemble with specific compounds into nanoparticles. After targeting macrophages, the supramolecules can block the mutual binding of macrophage SIRP-α receptors and CD47, and block the CSF-1R signaling pathway, so that TAMs are polarized to the M1 type and induce an innate immune response against tumors 155.

**Figure 8:**
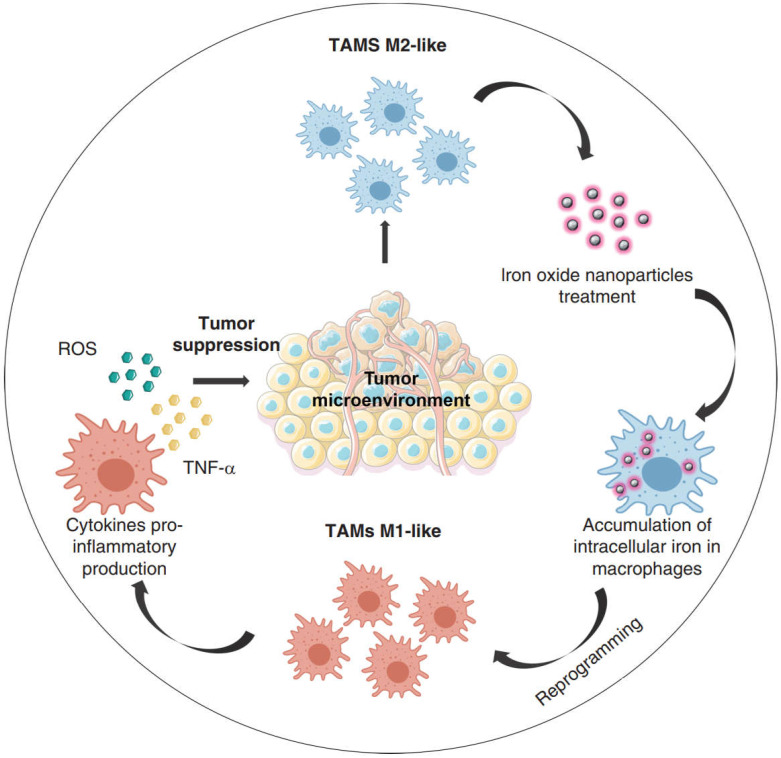
Reprogramming of tumor-associated macrophages by iron oxide nanoparticles as an anti-tumor therapeutic strategy 159. Copyright Year 2021, the Authors.

Several classes of microbial agents and microbe derived molecules (e.g., bacterial muramyl dipeptide, IFN- γ, CD40 antibody antagonist, CP-870, 893) were shown to drive the antitumor cytotoxic effects of macrophages, related clinical research has also been carried out, but their clinical efficacy has been modest 156,157. While the continuous extension of nanotechnology in the field of tumor immunotherapy has allowed many novel nanomedicines to be mined for their potential in tumor immunotherapy, iron oxide nanoparticles are one of them (**Figure 8**). For a long time, iron oxide nanoparticles have been used for imaging liver tumors due to the trait of specific aggregation in liver tissue, a property that highlights their advantages for liver tumor therapy 158,159. Li *et al*. used hyaluronic acid (HA) encapsulated superparamagnetic iron oxide nanoparticles (IONs) to construct reprogrammed macrophages (HION@Macs). *In vitro* and *in vivo* studies demonstrated that HION@Macs were able to educate *in situ* M2 macrophages toward M1 transition and restore their immune effects via a paracrine manner. HION@Macs also exhibits strong resistance to the immunosuppressive tumor environment, enabling sustained production of inflammatory factors (i.e., NO, H_2_O_2_, and TNF-α) to inhibit the growth of cancer cells and promote their apoptosis, exerting a synergistic tumor suppressive effect 160.

Although there have been many studies that successfully induced phenotypic transformation of macrophages *in vivo*, this strategy is still very far from clinical translation. Because in most cases, M1 type macrophages induced to undergo polarization by *in vitro* stimulation readily revert to the M2 phenotype after reaching the tumor immune microenvironment 161, thereby failing to generate sustained immune killing. Therefore, to truly exert powerful therapeutic effects in the clinic, macrophage-based tumor immunotherapy must develop effective strategies to control the phenotype of adoptively transferred macrophages *in vivo*. Based on this purpose, a cellular “backpack” strategy that can maintain the duration of M1type polarization of macrophages has emerged. Several drug delivery demonstrations using the “backpack” strategy have been carried out over the past few years 162-166.

Wyatt Shields IV *et al*. integrated interferon gamma (IFN-γ) into a “backpack”, and IFN-γ as a pro-inflammatory macrophage stimulator can sustain macrophages in a tumor-killing M1 type state. In mice with cancer, the maintenance effect slowed tumor growth and reduced metastasis for up to five days. Specifically, the team fabricated two layers of the biocompatible polymer poly (lactic-co-glycolic acid, PLGA) sandwiched with polyvinyl alcohol (PVA) and the cytokine IFN-γ. To complete the “backpack”, a layer of cellular adhesive was also added to help the “backpack” fit snugly on the macrophages. In addition, they attach easily and do not become phagocytosed or digested by macrophages due to their disc-shape, allowing them to survive for longer periods of time. The researchers injected “backpack”-carrying M1 macrophages into the tumors of mice and assessed them seven days later. Based on the findings of the *in vivo* study, macrophages carrying an IFN-γ “backpack” exhibited M1 indicators for more than 48 hours. As compared to cells injected with free IFN-γ or cells with a blank “backpack”, there was a significant difference in expression level of M1 indicators. Additionally, treatment with IFN-γ “backpack” therapy resulted in fewer metastatic nodules, smaller tumors, and longer lifespans than control group. Interestingly, not only do “backpack” -wearing macrophages stay in the M1 state, but it can also repolarize other macrophages including TAMs towards the M1 phenotype *in vivo*. In contrast to other studies, this effect was achieved at 100 times less than the maximum total dose of IFN-γ, and the mice did not show any signs of treatment toxicity 167.

As a result of the “backpack” strategy, macrophages adoptively transferred into solid tumors will retain their phenotype in an immunosuppressive environment. To improve its efficacy against solid tumors, future research can continue to delve into the optimal way of packing the stimulator into the “backpack” and its release kinetics. In addition, “backpack” can be combined with adjunctive therapies to increase the therapeutic effect. These strategies should also not be restricted to macrophages and can also engage other circulating cells with increased chemotactic sensitivity to mount a more robust tumor immune response 168,169.

## 4 Semi-biological hybrid cellular transformers

Abundant chemical groups on cell membranes are able to create interactions with the interfaces of nanomaterials or carriers. Based on this, coupling synthetic materials to the cell surface or encapsulating nano-drugs inside the cells can promote the exposure of tumor-associated antigens and induce tumor immune responses on the one hand, on the other hand can realize multimodal therapy of tumors by combining PDT or PTT, and most importantly, the constructed biomimetic nanocarriers have the property of actively targeting tumor sites. Such biomimetic nano-drug delivery systems constructed or modified artificially we call them semi-biological hybrid cellular transformers. By active targeting effect, it can reduce the non-specific distribution of drugs in the body, and the safety and efficacy of treatment can be improved 170. Through endocytosis mediated by the interaction of surface conjugating ligands with membrane receptors of target cells, semi-bio hybrid cellular transformer can increase the concentration of drugs inside the target cells 171. A series of nanocarriers modified with ligands, antibodies, antibody fragments or short peptides with high specificity and affinity have been developed so far according to the receptors or protein antigens that are highly expressed specifically on the tumor cell membrane 172. For example, transferrin can bind to transferrin receptors on the membranes of metastatic tumors and drug-resistant tumor cells to mediate endocytosis of drugs 173. It has been shown that Arginine glycine aspartic acid (RGD) tripeptides target tumor vessel and cell integrins αvβ3 that are upregulated on the surface of tumor cells and increase the delivery efficiency of drugs 174. In addition to protein ligands, carbohydrate ligands (e.g., hyaluronic acid, HA), folate receptors that are highly expressed in a variety of tumor cells, and nucleic acid aptamers can also bind receptors specific to tumor cell surfaces, conjugating them onto the nano-biomimetic drug delivery system not only mediates efficient uptake of drugs, but also enables enhanced delivery efficiency and safety of nanomedicines 175,176.

In addition to artificially modified ligands, certain specific types of cells have been regarded as potential drug delivery vehicles due to their abilities to homing to tumors and long circulation in the blood. For example, tumor cell membranes can be exploited for surface functionalization of nanoparticles, thereby endowing them with the ability for homologous targeting to achieve tumor specific drug delivery 177. Engineered CCMs prepared by fusing CCMs with other types of CCMs or making CCMs express specific proteins provide a new direction for overcoming the defects of CCMs and improving the biomedical properties of CCMs 178. The latest research points out that the nano-drug coated with glioblastoma (GBM) cell membrane has good blood-brain barrier (BBB) permeability and no obvious side effects 179. Besides, since a variety of immune cells are recruited to the tumor site during cancer development, this inspired researchers to use the cell membrane coated nanomedicine of immune cells to obtain the dual roles of tumor targeting and immunotherapy. It has been confirmed that nano-biomimetic drug delivery systems based on monocyte/macrophage membrane, neutrophil membrane and immune cell membrane can improve the delivery efficiency of antitumor drugs 180-183.

Although semi-biological hybrid cellular transformers can also be surface modified or loaded with drugs, there are some differences with EVs drug delivery systems. The difference between the two is that EVs, as part of the cell, only inherit some of the cell's properties. In order to develop dynamic drug delivery systems using EVs, it is usually necessary to introduce functional components into cells and release them in the form of EVs when the parent cells are exposed to specific stimuli 184. The preparation process is complex and the yield is difficult to control. However, the direct loading of drugs into the isolated EVs may lead to vesicle aggregation and membrane protein damage 185. In addition, the large-scale extraction and purification of EVs has been a major problem restricting their clinical application 186. In contrast, semi-biological hybrid cellular transformers mostly use cell membranes as carriers, so the types of surface proteins and markers are more abundant, and there are more possibilities in preparing targeted carriers. In addition, semi-biological hybrid cellular transformers can be prepared by using single-cell biofilms to encapsulate drugs, or by preparing hybrid membranes to impart more properties to the carriers. The more accessible membrane materials and higher carrier yield also make it more universal than EVs in the future. It is worth noting that compared with synthetic NPs, drug carriers constructed by natural cell membranes or EVs still have incomparable advantages. They can effectively load therapeutic drugs without significantly changing the membrane structure and surface protein, and have stability, biocompatibility, extremely low immunogenicity, targeting and the ability to overcome natural barriers in the blood circulation, which can overcome the limitations of synthetic NPs 187-189.

### 4.1 Cargo loaded cellular transformers

Loss of tumor antigen expression is one of the important mechanisms of immune escape. Tumor cells evade killing by the immune system by downregulating the expression of strong rejection antigens, downregulating major histocompatibility antigen I (MHC-I) levels, and impairing antigen-presenting function 190. Utilizing semi biological hybrid cellular transforms to deliver drugs specifically to tumor cells, and induce specific types of cell death, would lead to the release of tumor associated antigens (TAAs), which in turn be recognized by immune cells and activate antitumor immune responses 191.

**Figure 9:**
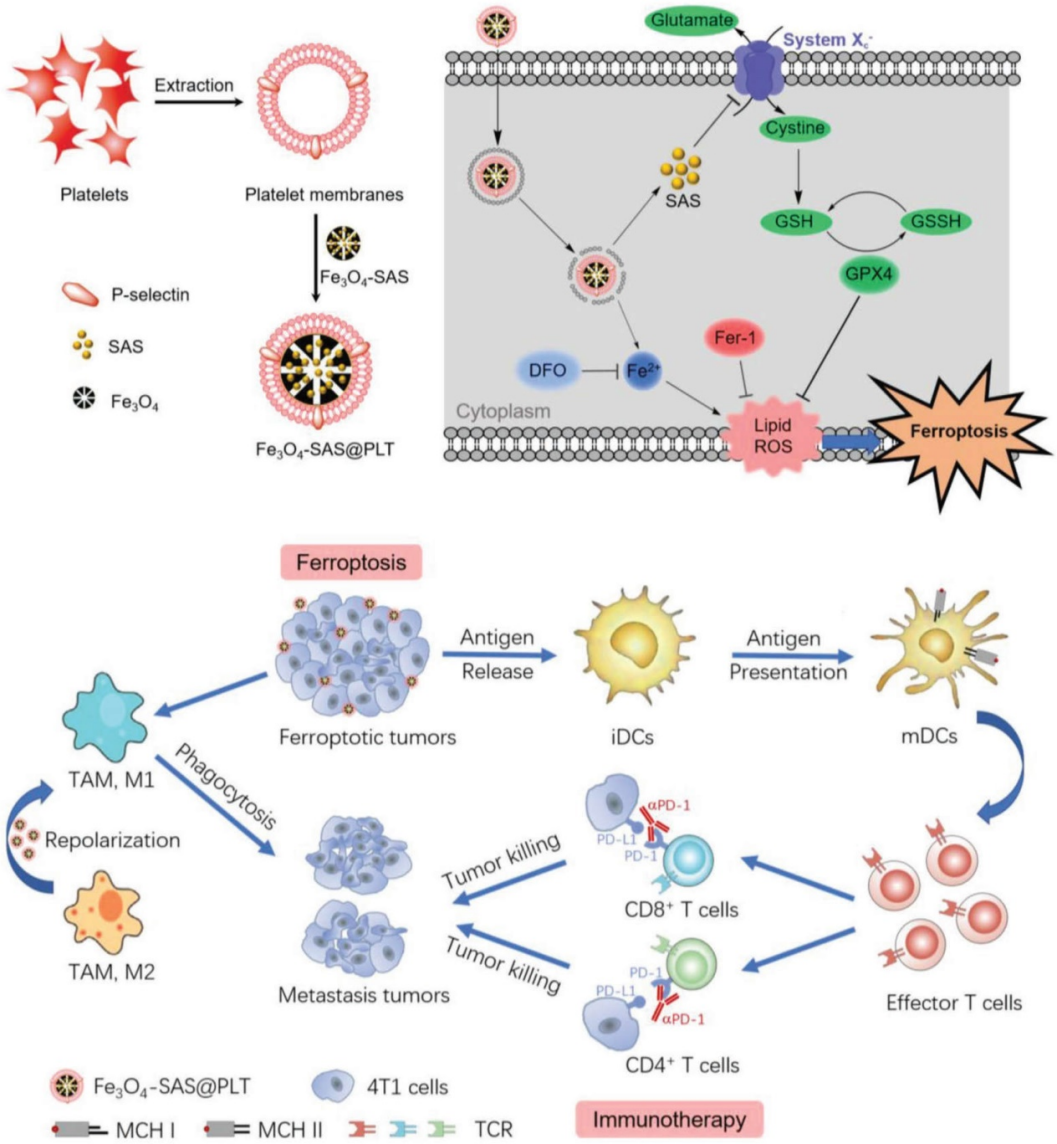
Schematic illustration of platelet membrane-camouflaged magnetic nanoparticles for ferroptosis-enhanced cancer immunotherapy. Adapted with permission from 192. Copyright Year 2020, Wiley-VCH GmbH.

Recently, a new form of cell death known as ferroptosis has been discovered that could improve immunogenicity of tumors. The existing agents that induce tumor cells to undergo ferroptosis usually face two major problems of being easily cleared by the body's immune system and being difficult to target. Jiang *et al*. coated sulfasalazine (SAS) - loaded magnetic iron oxide nanoparticles (Fe_3_O_4_ NP) with platelet membrane by extrusion method to obtain biomimetic nanoparticles Fe_3_O_4_- SAS@PLT with better stability. P-selectin on the surface of platelets works in conjunction with CD44 on the surface of 4T1 tumor cells to increase the uptake of nanoparticles and promote the accumulation of nanoparticles in metastatic tumors. The ingested magnetic iron oxide and sulfasalazine synergistically induce ferroptosis, expose TAAs, and activate antitumor immune responses (**Figure 9**). The rate of DC maturation induced by biomimetic nanocarriers was increased to 69.9%, which was significantly higher than that of nanoparticles without platelet membrane coating (28.4%). Programmed death (PD-1) inhibitors and this biomimetic nanocarrier were combined to treat tumor bearing mice, achieving 76% of mice survival up to 80 days, while the mice in other control groups all died within 50 days 192. This approach of utilizing natural ligands of cell membranes to deliver drugs specifically to tumor sites provides a feasible strategy to enhance antitumor immunity, meanwhile, the outstanding biocompatibility from cell membranes improves the possibility of clinical translation.

There are many tumors stromal cells in the TME, including cancer-associated fibroblasts (AF), and this occupies a major position as a barrier to the development of cancer treatments. On the one hand, AF secretes many growth factors and cytokines to activate tumor related signaling pathways to promote angiogenesis, tumorigenesis, progression, metastasis, and resistance generation 193. On the other hand, AF prevents drug entry into tumor cells by surrounding tumor cells and producing extracellular matrix (ECM) 194. It is illustrated that AF constitutes a physical barrier to protect tumor cells and impedes the access of anticancer drugs to the tumor to exert their effects. Many studies have therefore turned to the treatment of tumors based on AF. Li *et al*. reported an activated AF coated semiconductor polymer nanoparticle (SPN) for enhanced tumor phototherapy 195. This nanocomplex, called AF-SPN, is constructed from a semiconductor polymer (SP) containing NIR absorption and the AF cell membrane. As a theranostic agent, SP can be used for both imaging and photothermal therapy by producing NIR fluorescence and singlet oxygen. By coating the AF cell membrane with NPs, homologous targeting was enabled, and promoting NPs accumulation in the vicinity of cancer cells and thus enhancing the efficacy of photodiagnostic therapy. Furthermore, this biomimetic nanocomposite can also be loaded with other drugs and imaging agents to provide a multimodal theranostic platform, providing new ideas for tumor theranostics.

### 4.2 Engineered modified cellular transformers

**Figure 10:**
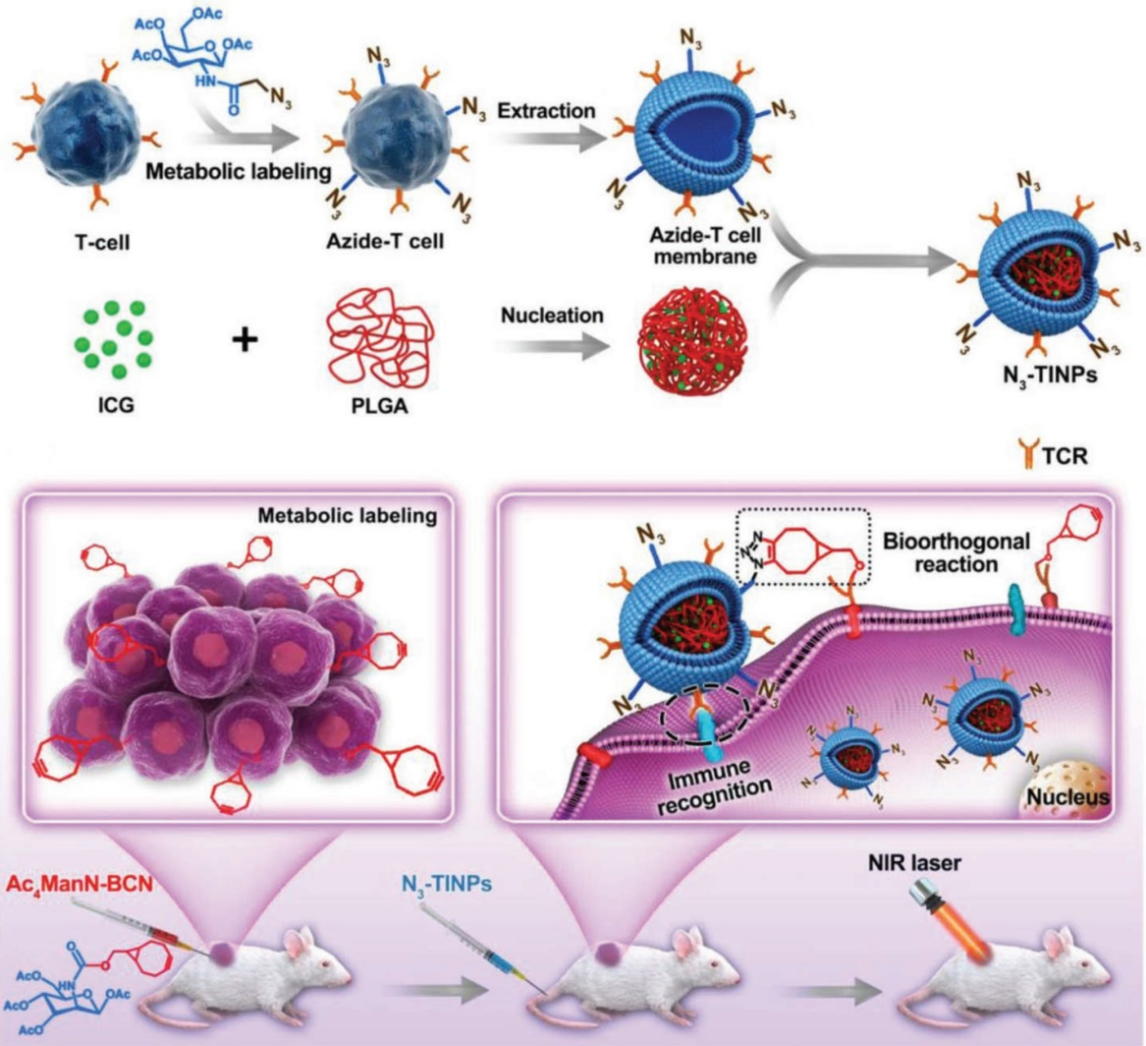
Schematic illustration of N3-labeled T cell membrane-biomimetic nanoparticles with dual-targeting mechanism for highly efficient photothermal therapy. Adapted with permission from 198. Copyright Year 2019, Wiley-VCH GmbH.

Recently, as a representative of adoptive cell therapy, CAR-T has achieved a large breakthrough in the treatment of hematological tumors. But due to the limitation of many factors, such as the lack of tumor specific antigen and the suppressive tumor immune microenvironment, CAR-T therapy still faces a great challenge in the treatment of solid tumors 196. In view of this, to improve CAR-T cell targeting and antitumor efficacy, we must start with optimization of CAR-T target selection, reduction of antigen escape and breaking immunosuppression in the TME 197. Owing to the high mutability of tumor cells and the changing immune microenvironment, the phenomenon of tumor immune escape is difficult to eliminate, and the dual targeting strategy can provide a more reliable and effective way for nanocarriers to target tumors. Researchers have used bioorthogonal technology to construct artificial targets BCN groups (bicyclic [6.1.0] nonyne) at the tumor site. Meanwhile, azide groups (N_3_) were embedded into T cell membranes by glycometabolism technology, and then the extracted N_3_ functionalized T cell membranes were encapsulated on the surface of indocyanine green (ICG) - loaded polymeric nanocores to construct biomimetic nanoparticles (N_3_⁃TINPs). A special protein (T-cell antigen receptor, TCR) on the T-cell membrane endowed N_3_⁃TINPs with immune recognition ability to tumor cells, while functionalized N_3_ groups enabled efficient and specific bioorthogonal reaction with BCN groups of artificial targets for glycometabolic labeling on tumor cells (**Figure 10**). N_3_⁃TINPs greatly enhance the enrichment of ICG at the tumor site to effectively overcome the tumor “off target effect”, thus improving the antitumor efficacy of nanocarriers based on their tumor targeting with immunomodulatory drugs using artificial targeting and natural target based dual guide therapy 198.

In recent years, aptamers as novel targeting ligands can also modulate the body's antitumor immune response by binding immune related receptors, which could be applied to tumor immunotherapy. In 2019, an osteosclerotic protein aptamer drug developed by the research team of Hong Kong Baptist University has been granted orphan drug certification by FDA for the treatment of osteogenesis imperfecta 199. Previous studies have shown that aptamers can be directly used in tumor immunotherapy as novel targeting ligands for tumor associated antigens, immune checkpoint molecules, costimulatory receptors, cytokines, and can also be coupled with drugs, siRNA, nanomaterials 200-202. Or modify the aptamer on the cell membrane surface to exert anti-tumor effect 203. The interaction between immune and tumor cells is an important link to achieve highly effective immunotherapy. By recognizing tumor cells on the surface of immune cell membranes, anchoring nucleic acid aptamers can directly induce the immune cells to attack the tumor cells and maximize the therapeutic effect. For example, the aptamer S4F targeting SGC-7901 gastric cancer cells were modified on the surface of CD3^+^T cells by metabolic labeling and click chemistry, giving them stronger tumor targeting and killing 204. NK cells modified with TLS11a aptamer and programmed cell death ligand 1 (PD-L1) aptamer can both target attacking liver cancer cells and resist the inhibition of PD-1/PD-L1 immune checkpoint, enhancing the immunotherapeutic efficacy of NK cells in solid tumors 205. Recently, researchers used DNA aptamers as a substitute for antibodies to construct a polyvalent antibody mimic (PAM), which utilizes hydrophobic insertion to insert the PAM into the cell membrane of NK cells to make NK cells identify and capture the corresponding tumor cells effectively. This method has a broad spectrum, and any aptamer of interest can be modified on various immune cells 206. Compared with CAR-T/NK immunotherapy, which has a large therapeutic risk and complicated operation, the aptamer modified immune cells are more easily available and have less toxic and side effects, making them highly potential for clinical applications.

Since tumor surface markers are different, the selection of suitable cell membrane surface modifiers is crucial for the targeting of biomimetic nanocarriers. Low density lipoprotein receptor related protein (LRP) receptors are overexpressed in endothelial cells of the BBB and in U87MG GBM cells. Based on this property, Shi *et al.* constructed an angiopep2 modified biomimetic nanocarrier of RBCs membrane loaded with polymer coloaded DOX and lexiscan (Lex). Lex can transiently open the BBB and increase the permeability of the nanomedicine to the brain, while angiopep2 can target the highly expressed LRP in the GBM, promoting the passage of the nanomedicine through BBB and achieving GBM targeting while prolonging the circulation time of the NPs 207. After further research, Shi *et al*. constructed ApoE peptide modified RBCs membrane encapsulated temozolomide (TMZ) and the epigenetic bromodomain inhibitor (OTX015) biomimetic nanocarriers to achieve synergistic chemotherapy and immune therapy for GL261 GBM. In contrast to previous studies, ApoE peptides can target multiple overexpressed low-density lipoprotein family receptors (LDLRs) (e.g., LDLR, LRP1, and LRP2) in BBB endothelial cells and GBM cells, thus providing superior BBB penetration and tumor targeting capabilities 208. This biomimetic nanocarrier enhances the sensitivity of TMZ to tumor cells while targeting GBM. The anti-tumor immune response was also enhanced by enhanced expression of CD4^ +^ and CD8^ +^ T cells through induction of immunogenic cell death and suppression of PD-1/PD-L1 coupling 209. Compared with brain tumors, digestive system tumors exist in a large variety and complex characteristics of biomarkers. Therefore, finding suitable targets is particularly important for the targeting, efficacy and safety of biomimetic nanocarriers.

### 4.3 Engineered cellular bioreactors

Currently, although surgery, chemotherapy, and radiotherapy are all effective methods of suppressing primary tumors, nearly 90% of cancer-related deaths are still caused by cancer cell metastasis 210. To improve cancer treatment outcomes, it may be beneficial to modulate the TME rather than directly destroy cancer cells 211. Studies have confirmed that the immunosuppressive TME of solid tumors is characterized by severe hypoxia, elevated H_2_O_2_ content, and overexpression of glutathione, while a cascade bioreactor constructed by combining natural cell membranes with nanomedicines is able to modulate the tumor microenvironment, improve radiotherapy, and support immunotherapy.

**Figure 11:**
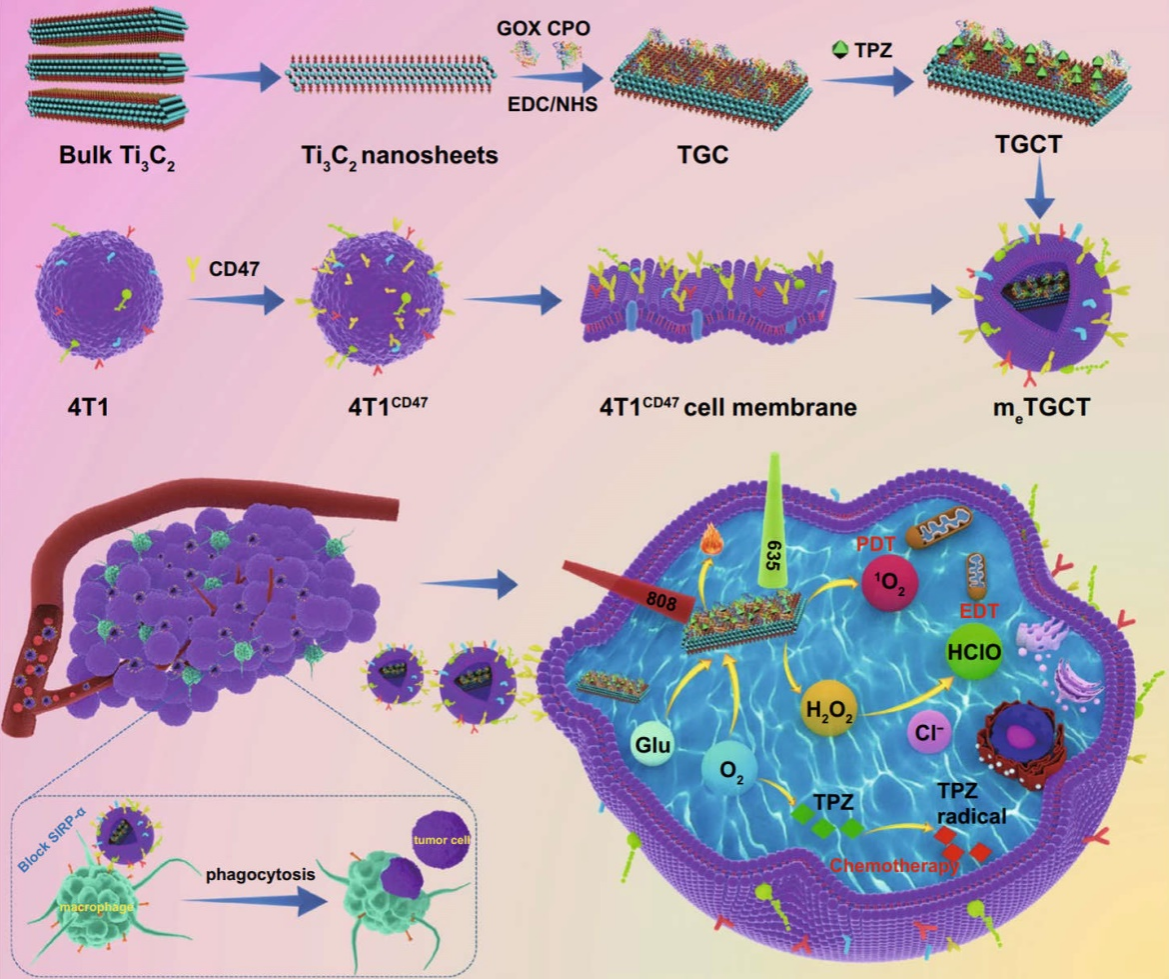
Schematic diagram of the construction of bionic cascaded-enzyme nanoreactor and proposed mechanism *in vivo*. Adapted with permission from 212. Copyright Year 2021, Springer Nature.

Liu *et al*. used Ti₃C₂ nanosheets as carriers to chemically conjugated with glucose oxidase (GOX) and chloroperoxidase (CPO) and load deoxygenation-activated prodrug tirapazamine (TPZ) to prepare a cascaded-enzyme nanoreactor Ti₃C₂- GOX - CPO / TPZ (TGCT), which was finally encapsulated into tumor cell membrane carriers with high expression of CD47 (mₑTGCT). Because the encapsulation of highly expressed CD47 biomimetic membrane, mₑTGCT exhibits superior immune escape ability as well as homologous targeting ability, which can be preferentially targeted to tumor sites and effectively enhanced tumor cell uptake. After entering tumor cells, GOX and CPO can produce a large amount of HClO through a cascade reaction, achieving highly efficient enzyme dynamic therapy (EDT). In addition, A further laser irradiation can increase singlet oxygen (¹O₂) production by accelerating enzyme catalyzed reactions. Meanwhile, the oxygen consumption of phototherapy and EDT could aggravate the hypoxic state inside the tumor, and then activate the deoxygenation-activated prodrug TPZ to realize chemotherapy. Experimental results showed that mₑTGCT can effectively inhibit tumor growth because of the amplified synergistic therapeutic effect of tumor phototherapy, enzyme dynamic therapy, and chemotherapy (**Figure 11**). A cascaded enzyme nanoreactor proves to be a promising way of achieving synchronized and remarkable antitumor therapy 212. At present, bacteria based synthetic biology has also been widely used in tumor therapy and achieved good results. Zhang *et al*. created a bacterium, *Escherichia coli* (MG1655), that overexpresses the NDH-2 enzyme (respiratory chain enzyme II) and colonizes tumors and increase the generation of H_2_O_2_. Subsequently, the experiments covalently linked magnetic Fe_3_O_4_ nanoparticles with bacteria and acted as a catalyst for Fenton-like reaction to transform H_2_O_2_ into toxic hydroxyl radical (•OH) for tumor therapy. The structures proved that the constructed bioreactor sustainably produced H_2_O_2_ with engineered bacteria for performing Fenton-like reaction, while the generated toxic •OH could effectively induce tumor cell apoptosis. With this bioreactor, it is possible to achieve aggressive tumor colonization as well as self-sufficient Fenton-like reactions without the need for additional H_2_O_2_ 213.

## Conclusions and future perspectives

Worldwide, digestive system cancer is still the leading cause of cancer death. Digestive system cancer is still the most common cause of cancer death worldwide. The clinical treatments for digestive system tumors are still conventional surgery, chemotherapy and radiotherapy. Surgical treatment, which is more effective for early-stage tumors that have not spread, but with the development and metastasis of malignant tumors, the curative effect and prognosis of advanced tumors are poor, and often cannot completely clear all cancer cells in the human body. Although chemotherapy can play a certain role, due to its lack of specificity, there is a large damage to the normal cells and immune system of the body, and it is easy to increase the body's drug resistance and cause adverse reactions. Radiotherapy has the problem of long cycle length and significant toxic side effects to human body. Some novel therapies such as immune checkpoint inhibitors, targeted agents, and CAR-T have played an important role in the treatment of digestive system cancer, but they also suffer from defects such as drug resistance and severe adverse effects. Under this background, the new idea of biomimetic nano-drug delivery system based on natural cells may change the situation of digestive system malignant tumor treatment (**Table 2**). The promotion effects of biomimetic nano-drug delivery systems for digestive system tumor therapy are mainly reflected in the following aspects. 1) The optimal multi-drug co-delivery system can be designed according to the inherent characteristics of various cell membranes, and the mixing ratio of cell membrane and nanoparticles can be adjusted to optimize the fusion process and achieve higher encapsulation efficiency and good controlled release capability. 2) Precise modification of nanocarriers can be achieved by recognition, removal or addition manipulation of specific antigenic proteins on the cell membrane surface to improve the tumor targeting ability. 3) By modifying nanocarriers with hybrid membranes, or incorporating ligands consisting of antibodies, nucleic acids or enzymes into the cell membrane for surface functionalization, a variety of biological functions beyond the single cell membrane are introduced to enhance the synergistic effect in tumor immunotherapy. 4) Cell membrane modified nanocarriers prepared from patients' own cells can be utilized for individualized drug delivery and treatment to achieve the effect of precision treatment of tumors. 5) It can promote the extensive combination of chemotherapy, radiotherapy, photothermal therapy and other treatment methods, and realize the integration of various tumor treatment methods.

Different from drug delivery for brain tumors, drug delivery systems targeting digestive system tumors can achieve targeted release of drugs without crossing complex biological barriers such as blood brain barrier. However, it should be noted that drug carriers targeting digestive system tumors still need to solve the problems such as short internal circulation time, off-target effect caused by being captured and cleared by immune system. In view of the low immunogenicity and high heterogeneity of digestive system tumors, the inherent targeting ability of immune cell membrane and the homologous targeting of tumor cell membrane can be used to achieve targeted drug release when designing natural cells based biomimetic cellular transformers. Compared with hematological tumors, the TME of the digestive system is relatively stable, and the infiltrating tumor associated macrophages are highly heterogeneous and plastic. Therefore, targeting the TME can relieve the immunosuppression of the tumor site, and transforming “cold tumors” into “hot tumors”. This is an effective strategy for designing biomimetic cellular transformers as well. Our review focuses on the dynamic properties of native cells and reviews their transformation characteristics. Targeting digestive system tumors by transferring specific functional and biological properties of native cells (such as dynamic deformation, phenotypic polarization, enhanced immunogenicity, immune escape, tumor-specific targeting) into nanocarriers treatment offers a wider range of possibilities.

Nonetheless, research on natural cell-based biomimetic nanoparticle drug delivery platforms in the diagnosis and treatment of digestive system tumors is still in its infancy, and many challenges need to be overcome for the transition from experimental research to clinical applications. One of the most important points is how to guarantee the safety of biomimetic NPs. Current production technologies of biomimetic NPs are not mature enough, and the resulting complex and varied production processes, inconsistent standards and poor reproducibility will affect the efficacy and safety of the final products; Second, existing characterization methods are limited, and the success of membrane coating is verified only by particle size detection and morphological observation. Western blot analysis can only demonstrate whether the surface composition of biomimetic nanocarriers is similar to that of the source cell membrane, but cannot verify whether the membrane is partially disrupted after membrane coating and whether biochemical reactions occur during the coating process or introduce unknown components that pose a hidden danger to drug safety. Moreover, biomimetic NPs with improper storage are prone to denaturation of membrane proteins, resulting in potential immune responses to endogenous antigens, which in turn cause tissue damage to the body. Additionally, its safety issue should be well considered when using certain special cell membranes such as tumor cell membrane or bacterial membrane. Inappropriate immunogenic exposure may induce detrimental immune responses, even against normal cell types, and long-term safety should be considered. Overall, to further improve the safety and efficacy of biomimetic NPs, the problem of poor reproducibility and inconsistent standards in the preparation process should first be addressed. Besides, innovative methods and infrastructure for natural cell acquisition require optimization, along with high-quality storage of natural cells and efficient characterization methods. During the production preparation process, it is imperative for the surface markers and specific functions of natural cells to not be destroyed. Yet, this remains an urgent problem in the current natural cell biomimetic nanoparticle drug delivery system towards clinical application. In addition, the tumor permeability, systemic cumulative toxicity, self-degradability and body excretion pathway after targeting biomimetic nanoparticle drug delivery to the tumor site still need to be explored.

The ultimate goal of discovering and developing biomimetic nano-drug delivery system is to reach the clinical stage and achieve large-scale industrial production. Most of the studies on the drug-loaded biomimetic NPs are still in the preclinical stage. Some formulations have undergone clinical research and may be used to serve patients soon (**Table 3**). In conclusion, despite the great success of natural cell biomimetic nanoparticle drug delivery for treating tumors, this innovative strategy still stays at the laboratory stage and cannot meet the requirements for clinical applications because there are currently no suitable methods to ensure the controllability of the preparation process and the safety and efficacy of the use process. However, challenges always coexist with opportunities. From the laboratory to the clinic, there are still many issues that must be addressed urgently. But the unique advantages and application potential of the natural cell biomimetic nanoparticle drug delivery system are still undeniable, which provides the design and application of nano-drugs. The new thinking mode will have a place in the field of precision medicine.

## Figures and Tables

**Table 1:** Major advantages and disadvantages of common EVs drug loading strategies

Drug loading methods	Advantages	Disadvantages	Ref
Freeze thaw	Medium drug loading efficiency and low cost	Protein degradation, irreversible change of EVs structure	80
Co-incubation	Simple operation, no large equipment required, no damage to membrane integrity	Affects the size of EVs, results in low yield, low entrapment and uncontrollable drug loading	81,82
Electroporation	Load macromolecular substances or hydrophilic molecules	Molecular aggregation, EVs structural instability	83
Extrusion	High drug loading efficiency	Destroy membrane integrity and alter membrane properties	84
Saponin assisted encapsulation	Highest drug loading efficiency	Presence of toxicity	85,86
Sonication	Suitable for biological molecules like small RNAs, high drug loading efficiency	Membrane dysregulation with incomplete drug release, non-suitability for hydrophobic drug delivery	82
Click chemistry	Fast and efficient, with strong binding site controllability	Potential Toxicity of Chemical Crosslinkers	82
Genetic engineering of parental cells	Allows the loading of RNA, DNA, and peptides of choice into the EVs	High cost and low efficiency, choice of specific EVs derived cells is an associated limitation	87,88
Noncovalent binding and Hydrophobic inlay	Can dissociate by changes in chemical shift, temperature, or solvent	Too costly to be synthesized in bulk	89,90
Microfluidics technology	Preservation of EVs integrity, stability maintenance, high throughput precision, and low sample volume	Sometimes polymeric material or non-EVs ingredient causes co-precipitation along with EVs	82

**Table 2:** The application of natural cell based biomimetic cellular transformers in the digestive system cancer

Cancer type	Nanomaterials	Sources of biomimetic materials	Cargo	Surface modifier	Target	Outcome/properties	Biosecurity	Ref
Esophageal Cancer	Poly (lactic-co-glycolic acid) (PLGA)	TE10 cells membrane	Doxorubicin/curcumin	DSPE-PEG15000	TE10/DOX xenografted tumor	Homologous targeting effect, synergistic anti-tumor effect	High biosafety	214
Esophageal Cancer	None	RBC membrane	Paclitaxel	Anti-EGFR-iRGD	EGFR extracellular domain, αυβ3 and neuropilin-1 receptors	Dual tumor-targeting strategy, improve the radiosensitization	Relatively safe concerning both manufacture and clinical use	215
Gastric cancer	Zeolitic imidazole frameworks-8	AGS cell	Chlorin e6/ tirapazamine	none	AGS tumor	Synergistic therapy of SDT and chemotherapy, induce pyroptosis of cancer cells and play anti-tumor effects	Excellent biocompatibility and biosafety	216
Gastric cancer	PLGA	RBC membrane	Paclitaxel/ triptolide	none	BGC-823/SGC-7901 cells	Evasion of immune surveillance and extension of blood circulation	none	217
Gastric cancer	none	RBC membrane	Paclitaxel	Injectable hydroge	MKN-45 tumor	Enhanced retention effect of nanoparticles in tumors	Low systemic toxicity	218
Gastric cancer	Amine SLN	SGC7901 cell membrane	Chlorins e6	none	SGC7901 tumor	Achieve sufficient PDT of gastric cancer	none	219
Gastric cancer	none	RBC membrane	Paclitaxel	DSPE-PEG-anti-EGFR-iRGD	MKN45 tumor	Enhance tumor-targeting ability, improve therapeutic efficiency of RBCm-PTX	No acute toxicity	220
Pancreatic cancer	PLGA	KPC cells membrane	Gemcitabine	M2pep peptide	Pancreatic orthotopic tumor	Targeting both tumor cells and M2-like TAMs, achieve effective combination of chemotherapy and immune checkpoint inhibitor therapy	Combinational therapy is biosafe	221
Pancreatic cancer	β-cyclodextrin	cancer cell-macrophage hybrid membrane	Gemcitabine/erlotinib/IRAK4; siRNA	none	PANC-1/ SW1990 tumor	*In vitro* synergistic anti-proliferation, anti-migration and pro-apoptosis effects	Excellent biocompatibility and biosafety	222
Pancreatic cancer	none	PATU-8988 cells membrane	PDEδ degrader	none	PATU-8988/ PL-45 cells	Induced cellular apoptosis and suppressed cell proliferation via the inhibition of RAS signaling	none	223
Pancreatic cancer	Gold nanorods	RBC membrane	Cyclopamine (oral administration)	none	Capan-2 tumor xenografts	Obtained excellent PTT efficacy	Combinational therapy is biosafe	224
Pancreatic cancer	PLGA	RBC membrane	Cyclopamine/ paclitaxel	none	Capan-2 tumor xenografts	Improved drug delivery to tumor, significantly enhance the anti-tumour efficacy	Combinational therapy is biosafe	225
Colorectal cancer	none	human platelet membrane	R848	none	MC38 tumor	Active tumor-specific T cell immune responses and long-term protective immunity	No acute toxicity	226
Colorectal cancer	PLGA	DC-MC38 fusion cell membrane	CpG ODN	none	MC38 tumor	A vaccine to prevent tumor development, a therapeutic agent to regress established tumors	Safe for *in vivo* use	227
Colorectal cancer	Zinc gallogermanate persistence luminescence nanomaterials	Erythrocyte -293T cell hybrid membrane	Cisplatin	PD-1	CT26 tumor	Excellent immune escapability and cancer active targeting ability	Excellent *in vitro* and *in vivo* biosafety	228
Colorectal cancer	none	leukocyte-derived membrane	Doxorubicin	none	HCT-116 organoids	Target the inflamed vasculature associated with colorectal cancer	none	229
Colorectal cancer	Bovine serum albumin/ Fe3O4	CT26 cancer cell membrane	Chlorin e6	none	CT26 tumor	Homologous targeting, combinational SDT and CDT could accelerate cell apoptosis	Excellent *in vivo* biosafety	230
Colorectal cancer	Mesoporous silica and zinc gallogermanate	Lactobacillus reuteri biofilm	5-FU	none	HT-29/MC-38 cells	Withstanding the digestion of gastric acid and targeted release 5-FU to colorectum	Good biocompatibility and no toxic effects on the primary organs	231
Liver cancer	Pectin	RBC membrane	Doxorubicin	none	BEL-7402 tumor	Prolong blood circulation, enhance active targeting	Low systemic toxicity,	232
Liver cancer	PLGA	RBC membrane	Plumbagin and Dihydrotanshi-none I	Mannose	Huh-7/Hepa1-6 tumor	Reverse the immunosuppressive TME, potentiate the systemic antitumor immunity	No significantly *in vivo* toxicity	233
Liver cancer	PLGA	HUVEC membrane	Oxaliplatin/ hydroxychloro-quine	TRAIL	Orthotopic HCCLM3 tumor/HepG2, Huh-7 or HCCLM3 metastatic tumor	Execute targeted autophagy inhibition, enhanced chemotherapy and antimetastatic effect	Low toxicity and excellent biocompatibility	234
Liver cancer	PLGA	RBC membrane	Doxorubicin/ indocyanine green	Folic acid	H22 tumor	Exert combinational antitumor effects of chemotherapy and thermotherapy	Satisfactory biocompatibility with no obvious cardiotoxicity	235
Liver cancer	Glyceryl monooleate/ P407	Huh-7 human cancer cell-platelet hybrid membrane	Sorafenib/ triptolide	none	Huh-7 tumor	Achieve long circulation function and tumor targeting, obtain a better "synergy and attenuation effect"	No significant toxicity	236
Liver cancer	Thermosensitive liposome	HepG2 cell membrane	Doxorubicin/ indocyanine green	none	HepG2 tumor	Induce the synergistic effect of photothermal and chemotherapy	Low toxicity and excellent biocompatibility	237
Liver cancer	Copper sulfide	H22 cancer cell-macrophage hybrid membrane	Sorafenib	Anti-VEGFR	H22 tumor	Achieve synergistic photo-thermal and chemotherapy	With minimal damage to the normal tissues	238
Liver cancer	Mesoporous silica	CAR-T cell membrane	IR780	GPC3-CAR	Huh-7 tumor	Exhibit photothermal antitumor abilities along with enhanced targeting abilities	Excellent biocompatibility and no short-term side effect	239
Liver cancer	PLGA	HepG2 cell membrane	Doxorubicin	none	HepG2 tumor	High stability, great immunocompatibility and excellent homotypic targeting ability	Low toxicity and excellent biocompatibility	240

**Table 3:** Clinical status of natural cell based biomimetic cellular transformers in the digestive system cancer

Conditions	Interventions/treatment	Carrier source	NCT number	Phase	Clinical Status
Advanced Esophageal Cancer	Anti-MUC1 CAR-T combined with PD-1 Knockout T cells	CAR-T cell	NCT03706326	Phase 1/2	Recruiting
Gastric Cancer	Biological: activated DCs	DCs membrane	NCT03410732	Phase 2	Recruiting
Pancreatic Cancer	Drug: GRASPA	RBCs	NCT01523808	Phase 1	Completed
Rectal Cancer	None	Tumor EVs	NCT04852653	None	Recruiting
colon cancer	Curcumin	Plant exosomes	NCT01294072	Phase 1	Recruiting
Colorectal Cancer	NKG2D CAR-NK	CAR-NK cell	NCT05213195	Phase 1	recruiting
Gastroesophageal Junction Cancer	N-803, Pembrolizumab, PD-L1 t-haNK	CAR-NK cell	NCT04847466	Phase 2	recruiting
Gastrointestinal Cancer	Cyclophosphamide, Fludarabine, Aldesleukin, Pembrolizumab	CAR-T cell	NCT03412877	Phase 2	recruiting
Solid Tumor	Cyclophosphamide	CAR-T cell	NCT02498912	Phase 1	Active, not recruiting
Solid Tumor	KITE-718, Cyclophosphamide, Fludarabine	CAR-T cell	NCT03139370	Phase 1	Active, not recruiting
Solid Tumor	ROBO1 CAR-NK cells	CAR-NK cell	NCT03940820	Phase 1/2	unknown
Advanced Solid Tumor	Anti-CAR-NK Cells	CAR-NK cell	NCT05194709	Early Phase 1	recruiting
Metastatic Malignant Solid Neoplasm	Atezolizumab, Cyclophosphamide, Fludarabine, PD1 Inhibitor	CAR-T cell	NCT04639245	Phase 2	Suspended
Metastatic Melanoma	Cyclophosphamide, Fludarabine, Aldesleukin	Tumor infiltrating lymphocytes	NCT01369875	Phase 2	Terminated

## Abbreviations

APCs: antigen-presenting cells
CTCs: circulating tumor cells
EVs: extracellular vesicles
MDSCs: myeloid-derived suppressor cells
NPs: nanoparticles
NETs: neutrophil extracellular traps
OMVs: outer membrane vesicles
PAMPs: pathogen-associated molecular patterns
PTT: photothermal therapy
PDENs: plant-derived exosome-like nanoparticles
PEG: polyethylene glycol
PM-NV: platelet-mimicking nanovehicles
PDT: photodynamic therapy
TAAs: tumor associated antigens
PD-1: programmed death-1
PD-L1: programmed death ligand-1
RBCs: red blood cells
SIRP-α: signal regulatory protein-α
TME: tumor micro-environment
TAMs: tumor associated macrophages

## References

[1] Mizrahi J, Pant S (2020). Immunotherapy in Gastrointestinal Malignancies. Adv Exp Med Biol.

[2] Sung H, Ferlay J, Siegel RL, Laversanne M, Soerjomataram I, Jemal A (2021). Global Cancer Statistics 2020: GLOBOCAN Estimates of Incidence and Mortality Worldwide for 36 Cancers in 185 Countries. CA Cancer J Clin.

[3] Mehrling T (2015). Chemotherapy is getting 'smarter'. Future Oncol.

[4] Turkes F, Mencel J, Starling N (2020). Targeting the immune milieu in gastrointestinal cancers. J Gastroenterol.

[5] Barsouk A, Thandra KC, Saginala K, Rawla P, Barsouk A (2020). Chemical Risk Factors of Primary Liver Cancer: An Update. Hepat Med.

[6] Dolcetti R, De Re V, Canzonieri V (2018). Immunotherapy for Gastric Cancer: Time for a Personalized Approach?. Int J Mol Sci.

[7] Park JY, Herrero R (2021). Recent progress in gastric cancer prevention. Best Pract Res Clin Gastroenterol.

[8] Shi J, Kantoff PW, Wooster R, Farokhzad OC (2017). Cancer nanomedicine: progress, challenges and opportunities. Nat Rev Cancer.

[9] Wolfram J, Ferrari M (2019). Clinical Cancer Nanomedicine. Nano Today.

[10] Hu Y, Liu T, Li J, Mai F, Li J, Chen Y (2019). Selenium nanoparticles as new strategy to potentiate gammadelta T cell anti-tumor cytotoxicity through upregulation of tubulin-alpha acetylation. Biomaterials.

[11] Zhang N, Song J, Liu Y, Liu M, Zhang L, Sheng D (2019). Photothermal therapy mediated by phase-transformation nanoparticles facilitates delivery of anti-PD1 antibody and synergizes with antitumor immunotherapy for melanoma. J Control Release.

[12] Pannuzzo M, Esposito S, Wu LP, Key J, Aryal S, Celia C (2020). Overcoming Nanoparticle-Mediated Complement Activation by Surface PEG Pairing. Nano Lett.

[13] Baghban R, Roshangar L, Jahanban-Esfahlan R, Seidi K, Ebrahimi-Kalan A, Jaymand M (2020). Tumor microenvironment complexity and therapeutic implications at a glance. Cell Commun Signal.

[14] Dong J, Gao HL, Wang WQ, Yu XJ, Liu L (2021). Bidirectional and dynamic interaction between the microbiota and therapeutic resistance in pancreatic cancer. Biochim Biophys Acta Rev Cancer.

[15] Xue J, Zhao Z, Zhang L, Xue L, Shen S, Wen Y (2017). Neutrophil-mediated anticancer drug delivery for suppression of postoperative malignant glioma recurrence. Nat Nanotechnol.

[16] Simeone P, Bologna G, Lanuti P, Pierdomenico L, Guagnano MT, Pieragostino D (2020). Extracellular Vesicles as Signaling Mediators and Disease Biomarkers across Biological Barriers. Int J Mol Sci.

[17] Picca A, Beli R, Calvani R, Coelho-Junior HJ, Landi F, Bernabei R (2020). Older Adults with Physical Frailty and Sarcopenia Show Increased Levels of Circulating Small Extracellular Vesicles with a Specific Mitochondrial Signature. Cells-Basel.

[18] Picca A, Guerra F, Calvani R, Bucci C, Lo MM, Bentivoglio AR (2019). Mitochondrial Dysfunction and Aging: Insights from the Analysis of Extracellular Vesicles. Int J Mol Sci.

[19] You Y, Borgmann K, Edara VV, Stacy S, Ghorpade A, Ikezu T (2020). Activated human astrocyte-derived extracellular vesicles modulate neuronal uptake, differentiation and firing. J Extracell Vesicles.

[20] Chen C, Zhang Y, Chen Z, Yang H, Gu Z (2021). Cellular transformers for targeted therapy. Adv Drug Deliver Rev.

[21] Zhang B, Tang G, He J, Yan X, Fan K (2021). Ferritin nanocage: A promising and designable multi-module platform for constructing dynamic nanoassembly-based drug nanocarrier. Adv Drug Deliv Rev.

[22] Duan SM, Zhang YL, Gao YJ, Lyu LZ, Wang Y (2021). The Influence of Long-Term Dietary Intake of Titanium Dioxide Particles on Elemental Homeostasis and Tissue Structure of Mouse Organs. J Nanosci Nanotechnol.

[23] AlMusawi S, Ahmed M, Nateri AS (2021). Understanding cell-cell communication and signaling in the colorectal cancer microenvironment. Clin Transl Med.

[24] McMahon TJ (2019). Red Blood Cell Deformability, Vasoactive Mediators, and Adhesion. Front Physiol.

[25] Guizouarn H, Barshtein G (2020). Editorial: Red Blood Cell Vascular Adhesion and Deformability. Front Physiol.

[26] Zhang CY, Dong X, Gao J, Lin W, Liu Z, Wang Z (2019). Nanoparticle-induced neutrophil apoptosis increases survival in sepsis and alleviates neurological damage in stroke. Sci Adv.

[27] Chu D, Dong X, Shi X, Zhang C, Wang Z (2018). Neutrophil-Based Drug Delivery Systems. Adv Mater.

[28] Zhang C, Zhang L, Wu W, Gao F, Li RQ, Song W (2019). Artificial Super Neutrophils for Inflammation Targeting and HClO Generation against Tumors and Infections. Adv Mater.

[29] Kuhn V, Diederich L, Keller TT, Kramer CM, Luckstadt W, Panknin C (2017). Red Blood Cell Function and Dysfunction: Redox Regulation, Nitric Oxide Metabolism, Anemia. Antioxid Redox Signal.

[30] Ayi K, Lu Z, Serghides L, Ho JM, Finney C, Wang J (2016). CD47-SIRPalpha Interactions Regulate Macrophage Uptake of Plasmodium falciparum-Infected Erythrocytes and Clearance of Malaria *In vivo*. Infect Immun.

[31] Rao L, Bu LL, Xu JH, Cai B, Yu GT, Yu X (2015). Red Blood Cell Membrane as a Biomimetic Nanocoating for Prolonged Circulation Time and Reduced Accelerated Blood Clearance. Small.

[32] Li C, Yang XQ, An J, Cheng K, Hou XL, Zhang XS (2020). Red blood cell membrane-enveloped O2 self-supplementing biomimetic nanoparticles for tumor imaging-guided enhanced sonodynamic therapy. Theranostics.

[33] Duez J, Carucci M, Garcia-Barbazan I, Corral M, Perez O, Presa JL (2018). High-throughput microsphiltration to assess red blood cell deformability and screen for malaria transmission-blocking drugs. Nat Protoc.

[34] Xia Q, Zhang Y, Li Z, Hou X, Feng N (2019). Red blood cell membrane-camouflaged nanoparticles: a novel drug delivery system for antitumor application. Acta Pharm Sin B.

[35] Soslau G (2020). The role of the red blood cell and platelet in the evolution of mammalian and avian endothermy. J Exp Zool B Mol Dev Evol.

[36] Dmitrieva LA, Pivovarov YI, Kurilskaya TE, Sergeeva AS (2016). Modern state of problem of delivery of medicines with use of erythrocytes as cell-carriers. Patol Fiziol Eksp Ter.

[37] Della PG, Kostevsek N (2021). Nucleic Acid Delivery with Red-Blood-Cell-Based Carriers. Int J Mol Sci.

[38] Tzounakas VL, Karadimas DG, Papassideri IS, Seghatchian J, Antonelou MH (2017). Erythrocyte-based drug delivery in Transfusion Medicine: Wandering questions seeking answers. Transfus Apher Sci.

[39] Zhang C, He T, Vedadghavami A, Bajpayee AG (2020). Avidin-biotin technology to synthesize multi-arm nano-construct for drug delivery. MethodsX.

[40] Magnani M, Chiarantini L, Mancini U (1994). Preparation and characterization of biotinylated red blood cells. Biotechnol Appl Biochem.

[41] Marczak A, Bukowska B (2013). ROS production and their influence on the cellular antioxidative system in human erythrocytes incubated with daunorubicin and glutaraldehyde. Environ Toxicol Pharmacol.

[42] Lotero LA, Olmos G, Diez JC (2003). Delivery to macrophages and toxic action of etoposide carried in mouse red blood cells. Biochim Biophys Acta.

[43] Brenner JS, Pan DC, Myerson JW, Marcos-Contreras OA, Villa CH, Patel P (2018). Red blood cell-hitchhiking boosts delivery of nanocarriers to chosen organs by orders of magnitude. Nat Commun.

[44] Ukidve A, Zhao Z, Fehnel A, Krishnan V, Pan DC, Gao Y (2020). Erythrocyte-driven immunization via biomimicry of their natural antigen-presenting function. Proc Natl Acad Sci U S A.

[45] Villa CH, Pan DC, Zaitsev S, Cines DB, Siegel DL, Muzykantov VR (2015). Delivery of drugs bound to erythrocytes: new avenues for an old intravascular carrier. Ther Deliv.

[46] Villa CH, Pan DC, Johnston IH, Greineder CF, Walsh LR, Hood ED (2018). Biocompatible coupling of therapeutic fusion proteins to human erythrocytes. Blood Adv.

[47] Brenner JS, Mitragotri S, Muzykantov VR (2021). Red Blood Cell Hitchhiking: A Novel Approach for Vascular Delivery of Nanocarriers. Annu Rev Biomed Eng.

[48] Ye H, Shen Z, Wei M, Li Y (2021). Red blood cell hitchhiking enhances the accumulation of nano- and micro-particles in the constriction of a stenosed microvessel. Soft Matter.

[49] Fan M, Jiang M (2020). Core-shell nanotherapeutics with leukocyte membrane camouflage for biomedical applications. J Drug Target.

[50] Li M, Li S, Zhou H, Tang X, Wu Y, Jiang W (2020). Chemotaxis-driven delivery of nano-pathogenoids for complete eradication of tumors post-phototherapy. Nat Commun.

[51] Galdiero MR, Bianchi P, Grizzi F, Di Caro G, Basso G, Ponzetta A (2016). Occurrence and significance of tumor-associated neutrophils in patients with colorectal cancer. Int J Cancer.

[52] Zhang Y, Hu Y, Ma C, Sun H, Wei X, Li M (2020). Diagnostic, Therapeutic Predictive, and Prognostic Value of Neutrophil Extracellular Traps in Patients with Gastric Adenocarcinoma. Front Oncol.

[53] Akk A, Springer LE, Yang L, Hamilton-Burdess S, Lambris JD, Yan H (2019). Complement activation on neutrophils initiates endothelial adhesion and extravasation. Mol Immunol.

[54] Michael M, Vermeren S (2019). A neutrophil-centric view of chemotaxis. Essays Biochem.

[55] Spiegel A, Brooks MW, Houshyar S, Reinhardt F, Ardolino M, Fessler E (2016). Neutrophils Suppress Intraluminal NK Cell-Mediated Tumor Cell Clearance and Enhance Extravasation of Disseminated Carcinoma Cells. Cancer Discov.

[56] Murphy PM (1997). Neutrophil receptors for interleukin-8 and related CXC chemokines. Semin Hematol.

[57] Demkow U (2021). Neutrophil Extracellular Traps (NETs) in Cancer Invasion, Evasion and Metastasis. Cancers (Basel).

[58] Kaltenmeier C, Simmons RL, Tohme S, Yazdani HO (2021). Neutrophil Extracellular Traps (NETs) in Cancer Metastasis. Cancers (Basel).

[59] Hellebrekers P, Vrisekoop N, Koenderman L (2018). Neutrophil phenotypes in health and disease. Eur J Clin Invest.

[60] Feng L, Dou C, Xia Y, Li B, Zhao M, Yu P (2021). Neutrophil-like Cell-Membrane-Coated Nanozyme Therapy for Ischemic Brain Damage and Long-Term Neurological Functional Recovery. Acs Nano.

[61] Li M, Li S, Zhou H, Tang X, Wu Y, Jiang W (2020). Chemotaxis-driven delivery of nano-pathogenoids for complete eradication of tumors post-phototherapy. Nat Commun.

[62] Zhang W, Huang X (2022). Stem cell membrane-camouflaged targeted delivery system in tumor. Materials Today Bio.

[63] Ferreira-Faria I, Yousefiasl S, Macário-Soares A, Pereira-Silva M, Peixoto D, Zafar H (2022). Stem cell membrane-coated abiotic nanomaterials for biomedical applications. J Control Release.

[64] Yang J, Lv K, Sun J, Guan J Anti-tumor effects of engineered mesenchymal stem cells in colon cancer model. 2019; 11: 8443-8450.

[65] Khosravi N, Pishavar E, Baradaran B, Oroojalian F, Mokhtarzadeh A (2022). Stem cell membrane, stem cell-derived exosomes and hybrid stem cell camouflaged nanoparticles: A promising biomimetic nanoplatforms for cancer theranostics. J Control Release.

[66] Zhang X, Sun Y, Chen W, Yang J, Chen J, Chen S (2022). Nanoparticle functionalization with genetically-engineered mesenchymal stem cell membrane for targeted drug delivery and enhanced cartilage protection. Biomater Adv.

[67] Liu Y, Zhao J, Jiang J, Chen F, Fang X Doxorubicin Delivered Using Nanoparticles Camouflaged with Mesenchymal Stem Cell Membranes to Treat Colon Cancer. 2020; 15: 2873-84.

[68] Yang N, Ding Y, Zhang Y, Wang B, Zhao X, Cheng K (2018). Surface Functionalization of Polymeric Nanoparticles with Umbilical Cord-Derived Mesenchymal Stem Cell Membrane for Tumor-Targeted Therapy. ACS Appl Mater Interfaces.

[69] Zhang M, Zhang F, Liu T, Shao P, Duan L, Yan J (2020). Polydopamine Nanoparticles Camouflaged by Stem Cell Membranes for Synergistic Chemo-Photothermal Therapy of Malignant Bone Tumors. Int J Nanomedicine.

[70] Kowal J, Arras G, Colombo M, Jouve M, Morath JP, Primdal-Bengtson B (2016). Proteomic comparison defines novel markers to characterize heterogeneous populations of extracellular vesicle subtypes. Proc Natl Acad Sci U S A.

[71] Allan D, Billah MM, Finean JB, Michell RH (1976). Release of diacylglycerol-enriched vesicles from erythrocytes with increased intracellular (Ca2+). Nature.

[72] Agrahari V, Agrahari V, Burnouf PA, Chew CH, Burnouf T (2019). Extracellular Microvesicles as New Industrial Therapeutic Frontiers. Trends Biotechnol.

[73] Shaimardanova AA, Solovyeva VV, Chulpanova DS, James V, Kitaeva KV, Rizvanov AA (2020). Extracellular vesicles in the diagnosis and treatment of central nervous system diseases. Neural Regen Res.

[74] Lange S (2021). Peptidylarginine deiminases and extracellular vesicles: prospective drug targets and biomarkers in central nervous system diseases and repair. Neural Regen Res.

[75] Marzano M, Bou-Dargham MJ, Cone AS, York S, Helsper S, Grant SC (2021). Biogenesis of Extracellular Vesicles Produced from Human-Stem-Cell-Derived Cortical Spheroids Exposed to Iron Oxides. Acs Biomater Sci Eng.

[76] Kang T, Atukorala I, Mathivanan S (2021). Biogenesis of Extracellular Vesicles. Subcell Biochem.

[77] Goh WJ, Zou S, Ong WY, Torta F, Alexandra AF, Schiffelers RM (2017). Bioinspired Cell-Derived Nanovesicles versus Exosomes as Drug Delivery Systems: a Cost-Effective Alternative. Sci Rep.

[78] Luan X, Sansanaphongpricha K, Myers I, Chen H, Yuan H, Sun D (2017). Engineering exosomes as refined biological nanoplatforms for drug delivery. Acta Pharmacol Sin.

[79] Villata S, Canta M, Cauda V (2020). EVs and Bioengineering: From Cellular Products to Engineered Nanomachines. Int J Mol Sci.

[80] Xi XM, Xia SJ, Lu R (2021). Drug loading techniques for exosome-based drug delivery systems. Pharmazie.

[81] Nasiri KA, Cheng L, Hill AF (2020). Methods for loading therapeutics into extracellular vesicles and generating extracellular vesicles mimetic-nanovesicles. Methods.

[82] Raghav A, Jeong GB (2021). A systematic review on the modifications of extracellular vesicles: a revolutionized tool of nano-biotechnology. J Nanobiotechnology.

[83] Lennaard AJ, Mamand DR, Wiklander RJ, El AS, Wiklander O (2021). Optimised Electroporation for Loading of Extracellular Vesicles with Doxorubicin. Pharmaceutics.

[84] Rayamajhi S, Aryal S (2020). Surface functionalization strategies of extracellular vesicles. J Mater Chem B.

[85] Hettich BF, Bader JJ, Leroux JC (2022). Encapsulation of Hydrophilic Compounds in Small Extracellular Vesicles: Loading Capacity and Impact on Vesicle Functions. Adv Healthc Mater.

[86] Haney MJ, Klyachko NL, Harrison EB, Zhao Y, Kabanov AV, Batrakova EV (2019). TPP1 Delivery to Lysosomes with Extracellular Vesicles and their Enhanced Brain Distribution in the Animal Model of Batten Disease. Adv Healthc Mater.

[87] Zhao L, Jiang X, Shi J, Gao S, Zhu Y, Gu T (2019). Exosomes derived from bone marrow mesenchymal stem cells overexpressing microRNA-25 protect spinal cords against transient ischemia. J Thorac Cardiovasc Surg.

[88] Limoni SK, Moghadam MF, Moazzeni SM, Gomari H, Salimi F (2019). Engineered Exosomes for Targeted Transfer of siRNA to HER2 Positive Breast Cancer Cells. Appl Biochem Biotechnol.

[89] Zhupanyn P, Ewe A, Buch T, Malek A, Rademacher P, Muller C (2020). Extracellular vesicle (ECV)-modified polyethylenimine (PEI) complexes for enhanced siRNA delivery *in vitro* and *in vivo*. J Control Release.

[90] Kwon S, Shin S, Do M, Oh BH, Song Y, Bui VD (2021). Engineering approaches for effective therapeutic applications based on extracellular vesicles. J Control Release.

[91] Li M, Jiang M, Meng J, Tao L (2019). Exosomes: Carriers of Pro-Fibrotic Signals and Therapeutic Targets in Fibrosis. Curr Pharm Des.

[92] Rufino-Ramos D, Albuquerque PR, Carmona V, Perfeito R, Nobre RJ, Pereira DAL (2017). Extracellular vesicles: Novel promising delivery systems for therapy of brain diseases. J Control Release.

[93] Willms E, Cabanas C, Mager I, Wood M, Vader P (2018). Extracellular Vesicle Heterogeneity: Subpopulations, Isolation Techniques, and Diverse Functions in Cancer Progression. Front Immunol.

[94] Meng W, He C, Hao Y, Wang L, Li L, Zhu G (2020). Prospects and challenges of extracellular vesicle-based drug delivery system: considering cell source. Drug Deliv.

[95] Weng Z, Zhang B, Wu C, Yu F, Han B, Li B (2021). Therapeutic roles of mesenchymal stem cell-derived extracellular vesicles in cancer. J Hematol Oncol.

[96] Kahmini FR, Shahgaldi S (2021). Therapeutic potential of mesenchymal stem cell-derived extracellular vesicles as novel cell-free therapy for treatment of autoimmune disorders. Exp Mol Pathol.

[97] Kok VC, Yu CC (2020). Cancer-Derived Exosomes: Their Role in Cancer Biology and Biomarker Development. Int J Nanomedicine.

[98] Yang P, Peng Y, Feng Y, Xu Z, Feng P, Cao J (2021). Immune Cell-Derived Extracellular Vesicles - New Strategies in Cancer Immunotherapy. Front Immunol.

[99] Lorenc T, Klimczyk K, Michalczewska I, Slomka M, Kubiak-Tomaszewska G, Olejarz W (2020). Exosomes in Prostate Cancer Diagnosis, Prognosis and Therapy. Int J Mol Sci.

[100] Wu HH, Zhou Y, Tabata Y, Gao JQ (2019). Mesenchymal stem cell-based drug delivery strategy: from cells to biomimetic. J Control Release.

[101] Zara M, Guidetti GF, Camera M, Canobbio I, Amadio P, Torti M (2019). Biology and Role of Extracellular Vesicles (EVs) in the Pathogenesis of Thrombosis. Int J Mol Sci.

[102] Silva AK, Luciani N, Gazeau F, Aubertin K, Bonneau S, Chauvierre C (2015). Combining magnetic nanoparticles with cell derived microvesicles for drug loading and targeting. Nanomedicine-Uk.

[103] Liang Y, Duan L, Lu J, Xia J (2021). Engineering exosomes for targeted drug delivery. Theranostics.

[104] Salunkhe S, Dheeraj, Basak M, Chitkara D, Mittal A (2020). Surface functionalization of exosomes for target-specific delivery and *in vivo* imaging & tracking: Strategies and significance. J Control Release.

[105] Li YJ, Wu JY, Liu J, Xu W, Qiu X, Huang S (2021). Artificial exosomes for translational nanomedicine. J Nanobiotechnology.

[106] Tran P, Xiang D, Tran T, Yin W, Zhang Y, Kong L (2020). Exosomes and Nanoengineering: A Match Made for Precision Therapeutics. Adv Mater.

[107] Tamura R, Uemoto S, Tabata Y (2017). Augmented liver targeting of exosomes by surface modification with cationized pullulan. Acta Biomater.

[108] Lee J, Lee H, Goh U, Kim J, Jeong M, Lee J (2016). Cellular Engineering with Membrane Fusogenic Liposomes to Produce Functionalized Extracellular Vesicles. ACS Appl Mater Interfaces.

[109] Ingato D, Lee JU, Sim SJ, Kwon YJ (2016). Good things come in small packages: Overcoming challenges to harness extracellular vesicles for therapeutic delivery. J Control Release.

[110] Dad HA, Gu TW, Zhu AQ, Huang LQ, Peng LH (2021). Plant Exosome-like Nanovesicles: Emerging Therapeutics and Drug Delivery Nanoplatforms. Mol Ther.

[111] Zhuang X, Deng ZB, Mu J, Zhang L, Yan J, Miller D (2015). Ginger-derived nanoparticles protect against alcohol-induced liver damage. J Extracell Vesicles.

[112] Bruno SP, Paolini A, D'Oria V, Sarra A, Sennato S, Bordi F (2021). Extracellular Vesicles Derived From Citrus sinensis Modulate Inflammatory Genes and Tight Junctions in a Human Model of Intestinal Epithelium. Front Nutr.

[113] Ju S, Mu J, Dokland T, Zhuang X, Wang Q, Jiang H (2013). Grape exosome-like nanoparticles induce intestinal stem cells and protect mice from DSS-induced colitis. Mol Ther.

[114] Deng Z, Rong Y, Teng Y, Mu J, Zhuang X, Tseng M (2017). Broccoli-Derived Nanoparticle Inhibits Mouse Colitis by Activating Dendritic Cell AMP-Activated Protein Kinase. Mol Ther.

[115] Raimondo S, Naselli F, Fontana S, Monteleone F, Lo DA, Saieva L (2015). Citrus limon-derived nanovesicles inhibit cancer cell proliferation and suppress CML xenograft growth by inducing TRAIL-mediated cell death. Oncotarget.

[116] Liu B, Lu Y, Chen X, Muthuraj PG, Li X, Pattabiraman M (2020). Protective Role of Shiitake Mushroom-Derived Exosome-Like Nanoparticles in D-Galactosamine and Lipopolysaccharide-Induced Acute Liver Injury in Mice. Nutrients.

[117] Cui Y, Gao J, He Y, Jiang L (2020). Plant extracellular vesicles. Protoplasma.

[118] Jisu K, Shiyi L, Shuya Z, Jianxin W (2022). Plant-derived exosome-like nanoparticles and their therapeutic activities. Asian J Pharm Sci.

[119] Wang B, Zhuang X, Deng ZB, Jiang H, Mu J, Wang Q (2014). Targeted drug delivery to intestinal macrophages by bioactive nanovesicles released from grapefruit. Mol Ther.

[120] Sun D, Zhuang X, Xiang X, Liu Y, Zhang S, Liu C (2010). A novel nanoparticle drug delivery system: the anti-inflammatory activity of curcumin is enhanced when encapsulated in exosomes. Mol Ther.

[121] Wu K, Xing F, Wu SY, Watabe K (2017). Extracellular vesicles as emerging targets in cancer: Recent development from bench to bedside. Biochim Biophys Acta Rev Cancer.

[122] Toyofuku M, Nomura N, Eberl L (2019). Types and origins of bacterial membrane vesicles. Nat Rev Microbiol.

[123] Rueter C, Bielaszewska M (2020). Secretion and Delivery of Intestinal Pathogenic Escherichia coli Virulence Factors via Outer Membrane Vesicles. Front Cell Infect Microbiol.

[124] Kaparakis-Liaskos M, Ferrero RL (2015). Immune modulation by bacterial outer membrane vesicles. Nat Rev Immunol.

[125] van der Pol L, Stork M, van der Ley P (2015). Outer membrane vesicles as platform vaccine technology. Biotechnol J.

[126] Kim OY, Park HT, Dinh N, Choi SJ, Lee J, Kim JH (2017). Bacterial outer membrane vesicles suppress tumor by interferon-gamma-mediated antitumor response. Nat Commun.

[127] Jain S, Pillai J (2017). Bacterial membrane vesicles as novel nanosystems for drug delivery. Int J Nanomedicine.

[128] Chen Q, Bai H, Wu W, Huang G, Li Y, Wu M (2020). Bioengineering Bacterial Vesicle-Coated Polymeric Nanomedicine for Enhanced Cancer Immunotherapy and Metastasis Prevention. Nano Lett.

[129] Li M, Zhou H, Yang C, Wu Y, Zhou X, Liu H (2020). Bacterial outer membrane vesicles as a platform for biomedical applications: An update. J Control Release.

[130] Brown GC (2019). The endotoxin hypothesis of neurodegeneration. J Neuroinflammation.

[131] Wang X, Thompson CD, Weidenmaier C, Lee JC (2018). Release of Staphylococcus aureus extracellular vesicles and their application as a vaccine platform. Nat Commun.

[132] Gujrati V, Kim S, Kim SH, Min JJ, Choy HE, Kim SC (2014). Bioengineered bacterial outer membrane vesicles as cell-specific drug-delivery vehicles for cancer therapy. Acs Nano.

[133] Geranpayehvaghei M, Dabirmanesh B, Khaledi M, Atabakhshi-Kashi M, Gao C, Taleb M (2021). Cancer-associated-platelet-inspired nanomedicines for cancer therapy. Wiley Interdiscip Rev Nanomed Nanobiotechnol.

[134] Holinstat M (2017). Normal platelet function. Cancer Metastasis Rev.

[135] Koupenova M, Kehrel BE, Corkrey HA, Freedman JE (2017). Thrombosis and platelets: an update. Eur Heart J.

[136] Taus F, Meneguzzi A, Castelli M, Minuz P (2019). Platelet-Derived Extracellular Vesicles as Target of Antiplatelet Agents. What Is the Evidence?. Front Pharmacol.

[137] Chen F, Liao Z, Peng D, Han L (2019). Role of Platelet Microparticles in Blood Diseases: Future Clinical Perspectives. Ann Clin Lab Sci.

[138] Haemmerle M, Stone RL, Menter DG, Afshar-Kharghan V, Sood AK (2018). The Platelet Lifeline to Cancer: Challenges and Opportunities. Cancer Cell.

[139] Contursi A, Grande R, Dovizio M, Bruno A, Fullone R, Patrignani P (2018). Platelets in cancer development and diagnosis. Biochem Soc Trans.

[140] Zmigrodzka M, Guzera M, Miskiewicz A, Jagielski D, Winnicka A (2016). The biology of extracellular vesicles with focus on platelet microparticles and their role in cancer development and progression. Tumour Biol.

[141] Zhao L, Bi Y, Kou J, Shi J, Piao D (2016). Phosphatidylserine exposing-platelets and microparticles promote procoagulant activity in colon cancer patients. J Exp Clin Cancer Res.

[142] Kanikarla-Marie P, Lam M, Menter DG, Kopetz S (2017). Platelets, circulating tumor cells, and the circulome. Cancer metastasis reviews.

[143] Best MG, Wesseling P, Wurdinger T (2018). Tumor-Educated Platelets as a Noninvasive Biomarker Source for Cancer Detection and Progression Monitoring. Cancer Res.

[144] Haschemi R, Gockel LM, Bendas G, Schlesinger M (2021). A Combined Activity of Thrombin and P-Selectin Is Essential for Platelet Activation by Pancreatic Cancer Cells. Int J Mol Sci.

[145] Wang H, Wu J, Williams GR, Fan Q, Niu S, Wu J (2019). Platelet-membrane-biomimetic nanoparticles for targeted antitumor drug delivery. J Nanobiotechnol.

[146] Hu Q, Sun W, Qian C, Wang C, Bomba HN, Gu Z (2015). Anticancer Platelet-Mimicking Nanovehicles. Adv Mater.

[147] Lu Y, Hu Q, Jiang C, Gu Z (2019). Platelet for drug delivery. Curr Opin Biotechnol.

[148] Ai X, Hu M, Wang Z, Zhang W, Li J, Yang H (2018). Recent Advances of Membrane-Cloaked Nanoplatforms for Biomedical Applications. Bioconjug Chem.

[149] Kailashiya J, Gupta V, Dash D (2019). Engineered human platelet-derived microparticles as natural vectors for targeted drug delivery. Oncotarget.

[150] Cohen R, Rousseau B, Vidal J, Colle R, Diaz LJ, Andre T (2020). Immune Checkpoint Inhibition in Colorectal Cancer: Microsatellite Instability and Beyond. Target Oncol.

[151] Gil DAC, Aleckovic M, Polyak K (2020). Immune Escape during Breast Tumor Progression. Cancer Immunol Res.

[152] Malfitano AM, Pisanti S, Napolitano F, Di Somma S, Martinelli R, Portella G (2020). Tumor-Associated Macrophage Status in Cancer Treatment. Cancers (Basel).

[153] Gunassekaran GR, Poongkavithai VS, Baek MC, Lee B (2021). M1 macrophage exosomes engineered to foster M1 polarization and target the IL-4 receptor inhibit tumor growth by reprogramming tumor-associated macrophages into M1-like macrophages. Biomaterials.

[154] Xiao H, Guo Y, Li B, Li X, Wang Y, Han S (2020). M2-Like Tumor-Associated Macrophage-Targeted Codelivery of STAT6 Inhibitor and IKKbeta siRNA Induces M2-to-M1 Repolarization for Cancer Immunotherapy with Low Immune Side Effects. ACS Cent Sci.

[155] Kulkarni A, Chandrasekar V, Natarajan SK, Ramesh A, Pandey P, Nirgud J (2018). A designer self-assembled supramolecule amplifies macrophage immune responses against aggressive cancer. Nat Biomed Eng.

[156] Xia Y, Rao L, Yao H, Wang Z, Ning P, Chen X (2020). Engineering Macrophages for Cancer Immunotherapy and Drug Delivery. Adv Mater.

[157] Cheng N, Bai X, Shu Y, Ahmad O, Shen P (2021). Targeting tumor-associated macrophages as an antitumor strategy. Biochem Pharmacol.

[158] Mulens-Arias V, Rojas JM, Barber DF (2021). The Use of Iron Oxide Nanoparticles to Reprogram Macrophage Responses and the Immunological Tumor Microenvironment. Front Immunol.

[159] Nascimento CS, Alves ÉAR, de Melo CP, Corrêa-Oliveira R, Calzavara-Silva CE (2021). Immunotherapy for cancer: effects of iron oxide nanoparticles on polarization of tumor-associated macrophages. Nanomedicine-Uk.

[160] Li CX, Zhang Y, Dong X, Zhang L, Liu MD, Li B (2019). Artificially Reprogrammed Macrophages as Tumor-Tropic Immunosuppression-Resistant Biologics to Realize Therapeutics Production and Immune Activation. Adv Mater.

[161] Lee S, Kivimae S, Dolor A, Szoka FC (2016). Macrophage-based cell therapies: The long and winding road. J Control Release.

[162] Klyachko NL, Polak R, Haney MJ, Zhao Y, Gomes Neto RJ, Hill MC (2017). Macrophages with cellular backpacks for targeted drug delivery to the brain. Biomaterials.

[163] Anselmo AC, Gilbert JB, Kumar S, Gupta V, Cohen RE, Rubner MF (2015). Monocyte-mediated delivery of polymeric backpacks to inflamed tissues: a generalized strategy to deliver drugs to treat inflammation. J Control Release.

[164] Xia J, Wang Z, Huang D, Yan Y, Li Y, Guan J (2015). Asymmetric Biodegradable Microdevices for Cell-Borne Drug Delivery. Acs Appl Mater Inter.

[165] Tang L, Zheng Y, Melo MB, Mabardi L, Castano AP, Xie YQ (2018). Enhancing T cell therapy through TCR-signaling-responsive nanoparticle drug delivery. Nat Biotechnol.

[166] Ayer M, Klok HA (2017). Cell-mediated delivery of synthetic nano- and microparticles. J Control Release.

[167] Shields CW, Evans MA, Wang LL, Baugh N, Iyer S, Wu D (2020). Cellular backpacks for macrophage immunotherapy. Sci Adv.

[168] Huang B, Abraham WD, Zheng Y, Bustamante LS, Luo SS, Irvine DJ (2015). Active targeting of chemotherapy to disseminated tumors using nanoparticle-carrying T cells. Sci Transl Med.

[169] Tang L, Zheng Y, Melo MB, Mabardi L, Castano AP, Xie YQ (2018). Enhancing T cell therapy through TCR-signaling-responsive nanoparticle drug delivery. Nat Biotechnol.

[170] Sun Q, Zhou Z, Qiu N, Shen Y (2017). Rational Design of Cancer Nanomedicine: Nanoproperty Integration and Synchronization. Adv Mater.

[171] Muhamad N, Plengsuriyakarn T, Na-Bangchang K (2018). Application of active targeting nanoparticle delivery system for chemotherapeutic drugs and traditional/herbal medicines in cancer therapy: a systematic review. Int J Nanomedicine.

[172] Harris JC, Scully MA, Day ES (2019). Cancer Cell Membrane-Coated Nanoparticles for Cancer Management. Cancers (Basel).

[173] Candelaria PV, Leoh LS, Penichet ML, Daniels-Wells TR (2021). Antibodies Targeting the Transferrin Receptor 1 (TfR1) as Direct Anti-cancer Agents. Front Immunol.

[174] Houdong Z (2019). iRGD: A Promising Peptide for Cancer Imaging and a Potential Therapeutic Agent for Various Cancers. J Oncol.

[175] Narmani A, Rezvani M, Farhood B, Darkhor P, Mohammadnejad J, Amini B (2019). Folic acid functionalized nanoparticles as pharmaceutical carriers in drug delivery systems. Drug Dev Res.

[176] Li X, Zhu X, Qiu L (2016). Constructing aptamer anchored nanovesicles for enhanced tumor penetration and cellular uptake of water soluble chemotherapeutics. Acta Biomater.

[177] Chen Z, Zhao P, Luo Z, Zheng M, Tian H, Gong P (2016). Cancer Cell Membrane-Biomimetic Nanoparticles for Homologous-Targeting Dual-Modal Imaging and Photothermal Therapy. Acs Nano.

[178] Guo Y, Wang Z, Shi X, Shen M (2022). Engineered cancer cell membranes: An emerging agent for efficient cancer theranostics. Exploration.

[179] Zou Y, Wang Y, Xu S, Liu Y, Yin J, Lovejoy DB (2022). Brain Co-Delivery of Temozolomide and Cisplatin for Combinatorial Glioblastoma Chemotherapy. Adv Mater.

[180] Kang T, Zhu Q, Wei D, Feng J, Yao J, Jiang T (2017). Nanoparticles Coated with Neutrophil Membranes Can Effectively Treat Cancer Metastasis. Acs Nano.

[181] Krishnamurthy S, Gnanasammandhan MK, Xie C, Huang K, Cui MY, Chan JM (2016). Monocyte cell membrane-derived nanoghosts for targeted cancer therapy. Nanoscale.

[182] Khatoon N, Zhang Z, Zhou C, Chu M (2022). Macrophage membrane coated nanoparticles: a biomimetic approach for enhanced and targeted delivery. Biomater Sci.

[183] Oroojalian F, Beygi M, Baradaran B, Mokhtarzadeh A, Shahbazi MA (2021). Immune Cell Membrane-Coated Biomimetic Nanoparticles for Targeted Cancer Therapy. Small.

[184] Sil S, Dagur RS, Liao K, Peeples ES, Hu G, Periyasamy P (2020). Strategies for the use of Extracellular Vesicles for the Delivery of Therapeutics. J Neuroimmune Pharmacol.

[185] Zhang LY, Yang X, Wang SB, Chen H, Pan HY, Hu ZM (2020). Membrane Derived Vesicles as Biomimetic Carriers for Targeted Drug Delivery System. Curr Top Med Chem.

[186] Ou YH, Liang J, Czarny B, Wacker MG, Yu V, Wang JW (2021). Extracellular Vesicle (EV) biohybrid systems for cancer therapy: Recent advances and future perspectives. Semin Cancer Biol.

[187] Guido C, Maiorano G, Cortese B, D'Amone S, Palama IE (2020). Biomimetic Nanocarriers for Cancer Target Therapy. Bioengineering (Basel).

[188] Tian X, Shi A, Wu J (2022). Construction of Biomimetic-Responsive Nanocarriers and their Applications in Tumor Targeting. Anticancer Agents Med Chem.

[189] Li A, Zhao Y, Li Y, Jiang L, Gu Y, Liu J (2021). Cell-derived biomimetic nanocarriers for targeted cancer therapy: cell membranes and extracellular vesicles. Drug Deliv.

[190] Shklovskaya E, Rizos H (2021). MHC Class I Deficiency in Solid Tumors and Therapeutic Strategies to Overcome It. Int J Mol Sci.

[191] Hsieh RC, Krishnan S, Wu RC, Boda AR, Liu A, Winkler M (2022). ATR-mediated CD47 and PD-L1 up-regulation restricts radiotherapy-induced immune priming and abscopal responses in colorectal cancer. Sci Immunol.

[192] Jiang Q, Wang K, Zhang X, Ouyang B, Liu H, Pang Z (2020). Platelet Membrane-Camouflaged Magnetic Nanoparticles for Ferroptosis-Enhanced Cancer Immunotherapy. Small.

[193] Santi A, Kugeratski FG, Zanivan S (2018). Cancer Associated Fibroblasts: The Architects of Stroma Remodeling. Proteomics.

[194] Huang Y, Zhou S, Huang Y, Zheng D, Mao Q, He J (2017). Isolation of Fibroblast-Activation Protein-Specific Cancer-Associated Fibroblasts. Biomed Res Int.

[195] Li J, Zhen X, Lyu Y, Jiang Y, Huang J, Pu K (2018). Cell Membrane Coated Semiconducting Polymer Nanoparticles for Enhanced Multimodal Cancer Phototheranostics. Acs Nano.

[196] Anurag KS, Joseph PM (2020). CAR T cells: continuation in a revolution of immunotherapy. The Lancet Oncology.

[197] Yang Y, McCloskey JE, Yang H, Puc J, Alcaina Y, Vedvyas Y (2021). Bispecific CAR T cells against EpCAM and inducible ICAM-1 overcome antigen heterogeneity and generate superior anti-tumor responses. Cancer Immunol Res.

[198] Han Y, Pan H, Li W, Chen Z, Ma A, Yin T (2019). T Cell Membrane Mimicking Nanoparticles with Bioorthogonal Targeting and Immune Recognition for Enhanced Photothermal Therapy. Adv Sci (Weinh).

[199] Zhang N, Zhang ZK, Yu Y, Zhuo Z, Zhang G, Zhang BT (2020). Pros and Cons of Denosumab Treatment for Osteoporosis and Implication for RANKL Aptamer Therapy. Front Cell Dev Biol.

[200] Li L, Xu S, Yan H, Li X, Yazd HS, Li X (2021). Nucleic Acid Aptamers for Molecular Diagnostics and Therapeutics: Advances and Perspectives. Angew Chem Int Ed Engl.

[201] Zhang Y, Lai BS, Juhas M (2019). Recent Advances in Aptamer Discovery and Applications. Molecules.

[202] He F, Wen N, Xiao D, Yan J, Xiong H, Cai S (2020). Aptamer-Based Targeted Drug Delivery Systems: Current Potential and Challenges. Curr Med Chem.

[203] Alamoudi AO (2021). Radiomics, aptamers and nanobodies: New insights in cancer diagnostics and imaging. Hum Antibodies.

[204] Liu CG, Wang Y, Liu P, Yao QL, Zhou YY, Li CF (2020). Aptamer-T Cell Targeted Therapy for Tumor Treatment Using Sugar Metabolism and Click Chemistry. Acs Chem Biol.

[205] Zhang D, Zheng Y, Lin Z, Liu X, Li J, Yang H (2020). Equipping Natural Killer Cells with Specific Targeting and Checkpoint Blocking Aptamers for Enhanced Adoptive Immunotherapy in Solid Tumors. Angew Chem Int Ed Engl.

[206] Shi P, Wang X, Davis B, Coyne J, Dong C, Reynolds J (2020). *In situ* Synthesis of an Aptamer-Based Polyvalent Antibody Mimic on the Cell Surface for Enhanced Interactions between Immune and Cancer Cells. Angew Chem Int Ed Engl.

[207] Zou Y, Liu Y, Yang Z, Zhang D, Lu Y, Zheng M (2018). Effective and Targeted Human Orthotopic Glioblastoma Xenograft Therapy via a Multifunctional Biomimetic Nanomedicine. Adv Mater.

[208] He W, Li X, Morsch M, Ismail M, Liu Y, Rehman FU (2022). Brain-Targeted Codelivery of Bcl-2/Bcl-xl and Mcl-1 Inhibitors by Biomimetic Nanoparticles for Orthotopic Glioblastoma Therapy. Acs Nano.

[209] Liu Y, Wang W, Zhang D, Sun Y, Li F, Zheng M (2022). Brain co-delivery of first-line chemotherapy drug and epigenetic bromodomain inhibitor for multidimensional enhanced synergistic glioblastoma therapy. Exploration.

[210] Chang M, Wang M, Wang M, Shu M, Ding B, Li C (2019). A Multifunctional Cascade Bioreactor Based on Hollow-Structured Cu_2_MoS4 for Synergetic Cancer Chemo-Dynamic Therapy/Starvation Therapy/Phototherapy/Immunotherapy with Remarkably Enhanced Efficacy. Adv Mater.

[211] Gong F, Chen M, Yang N, Dong Z, Tian L, Hao Y (2020). Bimetallic Oxide FeWOX Nanosheets as Multifunctional Cascade Bioreactors for Tumor Microenvironment-Modulation and Enhanced Multimodal Cancer Therapy. Adv Funct Mater.

[212] Zhang X, Cheng L, Lu Y, Tang J, Lv Q, Chen X (2022). A MXene-Based Bionic Cascaded-Enzyme Nanoreactor for Tumor Phototherapy/Enzyme Dynamic Therapy and Hypoxia-Activated Chemotherapy. Nano-Micro Lett.

[213] Fan JX, Peng MY, Wang H, Zheng HR, Liu ZL, Li CX (2019). Engineered Bacterial Bioreactor for Tumor Therapy via Fenton-Like Reaction with Localized H_2_O_2_ Generation. Adv Mater.

[214] Gao Y, Zhu Y, Xu X, Wang F, Shen W, Leng X (2021). Surface PEGylated Cancer Cell Membrane-Coated Nanoparticles for Codelivery of Curcumin and Doxorubicin for the Treatment of Multidrug Resistant Esophageal Carcinoma. Frontiers in Cell and Developmental Biology.

[215] Ren W, Sha H, Yan J, Wu P, Yang J, Li R (2018). Enhancement of radiotherapeutic efficacy for esophageal cancer by paclitaxel-loaded red blood cell membrane nanoparticles modified by the recombinant protein anti-EGFR-iRGD. J Biomater Appl.

[216] Yu Z, Cao W, Han C, Wang Z, Qiu Y, Wang J (2022). Biomimetic Metal-Organic Framework Nanoparticles for Synergistic Combining of SDT-Chemotherapy Induce Pyroptosis in Gastric Cancer. Frontiers in Bioengineering and Biotechnology.

[217] Wang S, Jiang H, Wang J, Wu H, Wu T, Ni M (2021). Superior *in vitro* anticancer effect of biomimetic paclitaxel and triptolide co-delivery system in gastric cancer. J Biomed Res.

[218] Qian H, Qian K, Cai J, Yang Y, Zhu L, Liu B (2019). Therapy for Gastric Cancer with Peritoneal Metastasis Using Injectable Albumin Hydrogel Hybridized with Paclitaxel-Loaded Red Blood Cell Membrane Nanoparticles. Acs Biomater Sci Eng.

[219] Yang J, Teng Y, Fu Y, Zhang C (2019). Chlorins e6 loaded silica nanoparticles coated with gastric cancer cell membrane for tumor specific photodynamic therapy of gastric cancer. Int J Nanomedicine.

[220] Chen H, Sha H, Zhang L, Qian H, Chen F, Ding N (2018). Lipid insertion enables targeted functionalization of paclitaxel-loaded erythrocyte membrane nanosystem by tumor-penetrating bispecific recombinant protein. Int J Nanomedicine.

[221] Wang M, Hu Q, Huang J, Zhao X, Shao S, Zhang F (2022). Engineered a dual-targeting biomimetic nanomedicine for pancreatic cancer chemoimmunotherapy. J Nanobiotechnol.

[222] Tang H, Xue Y, Li B, Xu X, Zhang F, Guo J (2022). Membrane-camouflaged supramolecular nanoparticles for co-delivery of chemotherapeutic and molecular-targeted drugs with siRNA against patient-derived pancreatic carcinoma. Acta Pharm Sin B.

[223] Fan R, He S, Wang Y, Qiao J, Liu H, Galstyan L (2022). Targeted delivery of a PROTAC induced PDEδ degrader by a biomimetic drug delivery system for enhanced cytotoxicity against pancreatic cancer cells. Am J Cancer Res.

[224] Jiang T, Zhang B, Shen S, Tuo Y, Luo Z, Hu Y (2017). Tumor Microenvironment Modulation by Cyclopamine Improved Photothermal Therapy of Biomimetic Gold Nanorods for Pancreatic Ductal Adenocarcinomas. Acs Appl Mater Inter.

[225] Jiang T, Zhang B, Zhang L, Wu X, Li H, Shen S (2018). Biomimetic nanoparticles delivered hedgehog pathway inhibitor to modify tumour microenvironment and improved chemotherapy for pancreatic carcinoma. Artif Cells Nanomed Biotechnol.

[226] Bahmani B, Gong H, Luk BT, Haushalter KJ, DeTeresa E, Previti M (2021). Intratumoral immunotherapy using platelet-cloaked nanoparticles enhances antitumor immunity in solid tumors. Nat Commun.

[227] Ma J, Liu F, Sheu WC, Meng Z, Xie Y, Xu H (2020). Copresentation of Tumor Antigens and Costimulatory Molecules via Biomimetic Nanoparticles for Effective Cancer Immunotherapy. Nano Lett.

[228] Wang Z, Liu J, Yang F, Hu Y, Lv H, Wang S (2021). Tailor-Made Cell-Based Biomimetic Nanoprobes for Fluorescence Imaging Guided Colorectal Cancer Chemo-immunotherapy. ACS Applied Bio Materials.

[229] Rampado R, Biccari A, D Angelo E, Collino F, Cricrì G, Caliceti P (2022). Optimization of Biomimetic, Leukocyte-Mimicking Nanovesicles for Drug Delivery Against Colorectal Cancer Using a Design of Experiment Approach. Frontiers in Bioengineering and Biotechnology.

[230] Chen X, Cheng D, Ding M, Yu N, Liu J, Li J (2022). Tumor-targeting biomimetic sonosensitizer-conjugated iron oxide nanocatalysts for combinational chemodynamic-sonodynamic therapy of colorectal cancer. J Mater Chem B.

[231] Wang Z, Liu J, Li C, Wang D, Lv H, Lv S (2019). Bacterial Biofilm Bioinspired Persistent Luminescence Nanoparticles with Gut-Oriented Drug Delivery for Colorectal Cancer Imaging and Chemotherapy. Acs Appl Mater Inter.

[232] Wang Y, Huang C, Ye P, Long J, Xu C, Liu Y (2022). Prolonged blood circulation outperforms active targeting for nanocarriers-mediated enhanced hepatocellular carcinoma therapy *in vivo*. J Control Release.

[233] Han S, Bi S, Guo T, Sun D, Zou Y, Wang L (2022). Nano co-delivery of Plumbagin and Dihydrotanshinone I reverses immunosuppressive TME of liver cancer. J Control Release.

[234] Shi Y, Lin G, Zheng H, Mu D, Chen H, Lu Z (2021). Biomimetic nanoparticles blocking autophagy for enhanced chemotherapy and metastasis inhibition via reversing focal adhesion disassembly. J Nanobiotechnol.

[235] Chen Z, Wang W, Li Y, Wei C, Zhong P, He D (2021). Folic Acid-Modified Erythrocyte Membrane Loading Dual Drug for Targeted and Chemo-Photothermal Synergistic Cancer Therapy. Mol Pharmaceut.

[236] Li Z, Yang G, Han L, Wang R, Gong C, Yuan Y (2021). Sorafenib and triptolide loaded cancer cell-platelet hybrid membrane-camouflaged liquid crystalline lipid nanoparticles for the treatment of hepatocellular carcinoma. J Nanobiotechnol.

[237] Sun Y, Zhai W, Liu X, Song X, Gao X, Xu K (2020). Homotypic cell membrane-cloaked biomimetic nanocarrier for the accurate photothermal-chemotherapy treatment of recurrent hepatocellular carcinoma. J Nanobiotechnol.

[238] Ji B, Cai H, Yang Y, Peng F, Song M, Sun K (2020). Hybrid membrane camouflaged copper sulfide nanoparticles for photothermal-chemotherapy of hepatocellular carcinoma. Acta Biomater.

[239] Ma W, Zhu D, Li J, Chen X, Xie W, Jiang X (2020). Coating biomimetic nanoparticles with chimeric antigen receptor T cell-membrane provides high specificity for hepatocellular carcinoma photothermal therapy treatment. Theranostics.

[240] Liu X, Sun Y, Xu S, Gao X, Kong F, Xu K (2019). Homotypic Cell Membrane-Cloaked Biomimetic Nanocarrier for the Targeted Chemotherapy of Hepatocellular Carcinoma. Theranostics.

